# Supplementary material for: Effect of UV-A on endophyte colonisation of Arabidopsis thaliana
Source: PLoS One. 2025 May 15;20(5):e0323576. doi: 10.1371/journal.pone.0323576 (PMC12080771; doi:10.1371/journal.pone.0323576)
Supplement: S1 Appendix — (DOCX) [file pone.0323576.s001.docx]

**Effect of UV-A on Endophyte Colonization of *Arabidopsis thaliana***

Aleksandra Giza, Paweł Hermanowicz, Rafał Ważny, Agnieszka Domka, Piotr Rozpądek, Justyna Łabuz

| **Organism** | **locus** | **Sequence (5’ – 3’)** | **Temp** | **nucleotides** |
| --- | --- | --- | --- | --- |
| *Paraphoma chrysanthemicola* | *TEF1* | GGGTTGTGCCGACTTTTTCG | 56°C | 20 |
|  |  | TTGAAGGAACCCTTGCCGAG |  | 20 |
| *Diaporthe eres* | *TEF1* | CATTTTCAGTGCGAGTGCGG | 60°C | 20 |
|  |  | GAAAGAGGCGATGGGGTTGA |  | 20 |
| *Mucor sp.,* | *TEF1* | GGTGATTCCGCTATCGTCAAG | 60°C | 20 |
|  |  | CCTTACCAGCCTTGTCAACC |  | 20 |
| *Sporobolomyces ruberrimus* | *Chitin synthase* | CAACGAGCGAACCCAAATCT | 53°C | 20 |
|  |  | TGCTCTGTTCTCCGACCTTT |  | 20 |
| *Phomopsis columnaris* | *TEF1* | GAGAAGGAAGGTTAGTAAA | 54°C | 19 |
|  |  | TTACCAAGCTCGGCGGCT |  | 18 |
| *Arabidopsis thaliana* | *Tubulin α-5, AT5G19780* | CCATATCCCCGTATCCATTT | 53°C-60°C | 20 |
|  |  | CATGTACTTTCCGTGCCTTG |  | 20 |

Table S1. Primer sequences used for qPCR confirming endophyte presence.

Table S2. Primer sequences used for real – time analysis of *Arabidopsis thaliana* gene expression.

| **gene** | **locus** | **Sequence (5’ – 3’)** | **Temp** | **nucleotides** |
| --- | --- | --- | --- | --- |
| *CRY1* | *AT4G08920* | AGGCATAGCGGTTCGATCAT | 56°C | 20 |
|  |  | ACACCAATGGATCCGCAACA |  | 20 |
| *CRY2* | *AT1G04400* | TGGTGGAACGTGGGATCTCT | 56°C | 20 |
|  |  | GAACACGCCCAAATCGCTTC |  | 20 |
| *UVR8* | *AT5G63860* | GTACAAATGGACAGCTCGGCAT | 56°C | 22 |
|  |  | TTACCTTTGCTTGAACCATCCG |  | 22 |
| *PDF1.2* | *At5g44420* | TCTTCGCTGCTCTTGTTCTC | 55°C | 20 |
|  |  | CTTGTGTGCTGGGAAGACAT |  | 20 |
| *ICS1* | *AT1G74710* | ATGAGATTCAGCCTCGCTGT | 55°C | 20 |
|  |  | TGATGGATCTCCAATCGTCA |  | 20 |
| *CHS1* | *AT5G13930* | TCTGGACACCAGACAGGACA | 55°C | 20 |
|  |  | GTAGTCAGCACCAGGCATGT |  | 20 |
| *PAL1* | *AT2G37040* | TGGATTCAAGGGAGCTGAGA | 55°C | 20 |
|  |  | TCAGAAGTTTTGCGAGACGA |  | 20 |
| *PHOT1* | *AT3G45780* | CACTGATCCTAGGCTTCCCG | 56°C | 20 |
|  |  | GTGGTTAGATCAGTCTCTGGACC |  | 23 |
| *PHOT2* | *AT5G58140* | GCTACCACTCTTGAGCGCATAGAG | 56°C | 24 |
|  |  | CCTCGCGTGAATACTCTGTC |  | 20 |
| *SAND* | *AT2G28390* | AACTCTATGCAGCATTTGATCCACT | 51°C | 25 |
|  |  | TGATTGCATATCTTTATCGCCATC |  | 24 |
| *PDF2* | *AT1G13320* | TAACGTGGCCAAAATGATGC | 51°C | 20 |
|  |  | GTTCTCCACAACCGCTTGGT |  | 20 |
