# Supplementary material for: Effect of UV-A on endophyte colonisation of Arabidopsis thaliana
Source: PLoS One. 2025 May 15;20(5):e0323576. doi: 10.1371/journal.pone.0323576 (PMC12080771; doi:10.1371/journal.pone.0323576)
Supplement: S2 Appendix — Tables S1-S7. (DOCX) [file pone.0323576.s002.docx]

**Effect of UV-A on Endophyte Colonization of *Arabidopsis thaliana***

Aleksandra Giza, Paweł Hermanowicz, Rafał Ważny, Agnieszka Domka, Piotr Rozpądek, Justyna Łabuz

Table S1. The results of the statistical analysis of the effects of the light conditions (two levels: only visible light /VIS/, visible light supplemented with UV-A /VIS + UVA/), the plant organ (two levels: roots, shoots) and inoculation status (two levels: inoculated vs non-inoculated) on the log-transformed ratio of fungal DNA to *Arabidopsis* DNA, measured with qPCR. Plants were inoculated with *Paraphoma chrysanthemicola* (A), *Phomopsis columnaris* (B), *Diaporthe eres* (C), *Mucor* sp. (D) or *Sporobolomyces ruberrimus* (E). A linear model of the relationship between the log-transformed endophyte DNA/plant DNA ratio and the predictors (light, organ, inoculation status, and their two- and three-way interactions) was fitted using the *gls* command of the *nlme* package in the R software. To control for heteroskedasticity, the model allowed for variance to differ between non-inoculated and inoculated samples (*nlme* variance function *varIdent(form = ~ 1|inoculationStatus)*). Differences between cell means of log-transformed responses were examined using the *emmeans* package, with the approximate number of degrees of freedom calculated using Satterthwaite’s method. *p* values were adjusted with Hommel’s method to keep the familywise error rate at 0.05. Four contrasts analyzed for the same endophyte were treated as a family for the purpose of the *p* adjustment.

**A**. Analysis of variance in the mean log-transformed ratio of *Paraphoma chrysanthemicola* DNA and *Arabidopsis* DNA levels

| Denominator df:18, numerator df: 1 | | | | |
| --- | --- | --- | --- | --- |
|  | *F* | *p* |  |  |
| organ | 9.4331 | 0.0066 | ** |  |
| light | 0.7408 | 0.4007 |  |  |
| inoculationStatus | 8.1191 | 0.0106 | * |  |
| organ:light | 0.6479 | 0.4314 |  |  |
| organ:inoculationStatus | 3.5343 | 0.0764 |  |  |
| light:inoculationStatus | 4.2246 | 0.0546 |  |  |
| organ:light:inoculationStatus | 0.0092 | 0.9248 |  |  |

Tests of differences in the mean log-transformed ratio of *Paraphoma chrysanthemicola* DNA and

*Arabidopsis* DNA levels

| Hypothesis | Estimate | SE | df | *t* | *p_adjusted_* |  |
| --- | --- | --- | --- | --- | --- | --- |
| roots:VIS: mock – inoculated = 0 | -2.60 | 2.00 | 11.80 | -1.30 | 0.4338 |  |
| shoots:VIS: mock – inoculated = 0 | 1.11 | 1.76 | 14.11 | 0.63 | 0.5373 |  |
| roots:(VIS + UVA): mock – inoculated = 0 | -6.29 | 1.76 | 14.11 | -3.58 | 0.0119 | * |
| shoots:(VIS+UVA): mock – inoculated = 0 | -2.93 | 2.00 | 11.80 | -1.47 | 0.3355 |  |

**B.** Analysis of variance of log-transformed ratio of *Phomopsis columnaris* DNA and *Arabidopsis*

DNA levels

| Denominator df:16, numerator df: 1 | | | |
| --- | --- | --- | --- |
|  | *F* | *p* |  |
| organ | 0.521 | 0.4808 |  |
| light | 1.297 | 0.2716 |  |
| inoculationStatus | 14.46 | 0.0016 | ** |
| organ:light | 0.674 | 0.4239 |  |
| organ:inoculationStatus | 17.30 | 0.0007 | *** |
| light:inoculationStatus | 0.232 | 0.6366 |  |
| organ:light:inoculationStatus | 0.845 | 0.3717 |  |

Tests of differences in the mean log-transformed ratio of *Phomopsis columnaris* DNA and

*Arabidopsis* DNA levels

| Hypothesis | Estimate | SE | df | *t* | *p_adjusted_* |  |
| --- | --- | --- | --- | --- | --- | --- |
| roots:VIS : mock – inoculated = 0 | -1.41 | 0.34 | 15.48 | -4.20 | 0.0029 | ** |
| shoots:VIS : mock – inoculated = 0 | 0.29 | 0.34 | 15.48 | 0.88 | 0.6090 |  |
| roots:(VIS + UVA): mock – inoculated = 0 | -1.26 | 0.34 | 15.48 | -3.76 | 0.0054 | ** |
| shoots:(VIS + UVA): mock – inoculated = 0 | -0.18 | 0.34 | 15.48 | -0.52 | 0.6090 |  |

**C.** Analysis of variance of the log-transformed ratio of *Diaphorte eres* DNA and *Arabidopsis* DNA

levels

| Denominator df:24, numerator df: 1 | | | |
| --- | --- | --- | --- |
|  | *F* | *p* |  |
| organ | 9.4875 | 0.0051 | ** |
| light | 0.0008 | 0.9782 |  |
| inoculationStatus | 179.62 | 1.23·10^-12^ | *** |
| organ:light | 0.0560 | 0.8149 |  |
| organ:inoculationStatus | 11.409 | 0.0025 | ** |
| light:inoculationStatus | 0.0096 | 0.9227 |  |
| organ:light:inoculationStatus | 8.7534 | 0.0068 | ** |

Tests of differences in the mean log-transformed ratio of *Diaphorte eres* DNA and *Arabidopsis* DNA

levels

| Hypothesis | Estimate | SE | df | *t* | *p_adjusted_* |  |
| --- | --- | --- | --- | --- | --- | --- |
| roots : VIS : mock – inoculated = 0 | -12.12 | 1.23 | 23.01 | -9.82 | 4.3·10^-9^ | *** |
| shoots : VIS : mock – inoculated = 0 | -4.30 | 1.23 | 23.01 | -3.48 | 0.0020 | ** |
| roots : (VIS + UVA): mock – inoculated = 0 | -8.59 | 1.23 | 23.01 | -6.96 | 1.28·10^-6^ | *** |
| shoots : (VIS + UVA): mock – inoculated = 0 | -8.08 | 1.23 | 23.01 | -6.54 | 2.25·10^-6^ | *** |

**D.** Analysis of variance of log-transformed ratio of *Mucor* sp. DNA and *Arabidopsis* DNA levels

| Denominator df:23, numerator df: 1 | | | |
| --- | --- | --- | --- |
|  | *F* | *p* |  |
| organ | 17.11 | 0.0004 | *** |
| light | 1.754 | 0.1984 |  |
| inoculationStatus | 10.78 | 0.0033 | ** |
| organ:light | 0.490 | 0.4910 |  |
| organ:inoculationStatus | 3.330 | 0.0810 |  |
| light:inoculationStatus | 0.390 | 0.5387 |  |
| organ: light:inoculationStatus | 0.307 | 0.5848 |  |

Tests of differences in the mean log-transformed ratio of *Mucor* sp. DNA and *Arabidopsis* DNA levels

| Hypothesis | Estimate | SE | df | *t* | *p_adjusted_* |  |
| --- | --- | --- | --- | --- | --- | --- |
| roots : VIS : mock – inoculated = 0 | -1.91 | 0.96 | 22.03 | -2.00 | 0.1756 |  |
| shoots : VIS : mock – inoculated = 0 | -0.68 | 1.02 | 22.88 | -0.66 | 0.5128 |  |
| roots : (VIS + UVA) : mock – inoculated = 0 | -3.06 | 0.96 | 22.03 | -3.19 | 0.0168 | * |
| shoots : (VIS + UVA) : mock – inoculated = 0 | -0.74 | 0.96 | 22.03 | -0.78 | 0.5128 |  |

**E.** Analysis of variance of log-transformed ratio of *Sporobomyces ruberrimus* DNA and *Arabidopsis* DNA levels

| Denominator df:24, numerator df: 1 | | | |
| --- | --- | --- | --- |
|  | *F* | *p* |  |
| organ | 15.860 | 0.0006 | *** |
| light | 0.5257 | 0.4754 |  |
| inoculationStatus | 116.84 | 1.05·10^-10^ | *** |
| organ : light | 0.4488 | 0.5093 |  |
| organ: inoculationStatus | 1.5440 | 0.2260 |  |
| light : inoculationStatus | 0.0043 | 0.9485 |  |
| organ : light:inoculationStatus | 0.1437 | 0.7080 |  |

Tests of differences in the mean log-transformed ratio of *Sporobomyces ruberrimus* DNA and

*Arabidopsis* DNA levels

| Hypothesis | Estimate | SE | df | *t* | *p_adjusted_* |  |
| --- | --- | --- | --- | --- | --- | --- |
| roots : VIS : mock – inoculated = 0 | -4.67 | 1.02 | 20.52 | -4.56 | 0.0002 | *** |
| shoots : VIS : mock – inoculated = 0 | -6.33 | 1.02 | 20.52 | -6.18 | 1.7·10^-5^ | *** |
| roots : (VIS + UVA) : mock – inoculated = 0 | -5.12 | 1.02 | 20.52 | -5.01 | 0.0001 | *** |
| shoots : (VIS + UVA) : mock – inoculated = 0 | -6.01 | 1.02 | 20.52 | -5.87 | 2.6·10^-5^ | *** |

Table S2. Results of the statistical analysis of the effects of endophyte inoculation (six levels, including the mock-inoculated control) and light conditions (two levels: only visible light /VIS/, visible light supplemented with UV-A /VIS + UVA/) on the following properties measured for Arabidopsis plants: root system length (A), average volume (B), average diameter (C), fresh weight of roots (D), fresh weight of shoots (E) and shoot anthocyanin content (F). A mixed linear model was fitted using the *lme* command of the *nlme* package in the R software. The model included fixed factors of endophyte and light conditions, as well as their interaction. The batch of plants was treated as a random intercept factor. To control for heteroskedasticity, the model allowed for different variance in each group (*nlme* variance function *varIdent(form = ~ 1| light*endophyte)*). The type III analysis of variance was performed using the *anova* command, with the effects coding of factor levels. Differences between cell means were examined using the *emmeans* package, with the approximate number of degrees of freedom calculated using Satterthwaite’s method. For the average root diameter, Satterthwaite’s method did not converged. A conservative number of degrees of freedom (smaller than provided with the containment method) was specified manually. *p* values are adjusted for the false discovery rate of 0.05 using the Benjamin-Hochberg method (within the family of 16 contrasts).

**A**. Analysis of variance of the root system length

|  | Num df | Den df | *F* | *p* | |  |
| --- | --- | --- | --- | --- | --- | --- |
| endophyte | 5 | 90 | 7.778 | 4·10^-6^ | *** | |
| light | 1 | 90 | 0.093 | 0.7609 |  | |
| endophyte:light | 5 | 90 | 0.602 | 0.6988 |  | |

Tests of differences in the mean root system length

| Hypothesis | Estimate [mm] | SE [mm] | df | *t* | *p_adjusted_* |  |
| --- | --- | --- | --- | --- | --- | --- |
| (VIS + UVA):*Mucor* – mock = 0 | -7.56 | 8.86 | 13.42 | -0.85 | 0.59453 |  |
| (VIS + UVA):*Diaphorte* - mock = 0 | -33.02 | 5.95 | 16.23 | -5.55 | 0.00066 | *** |
| (VIS + UVA):*Paraphoma* - mock = 0 | -18.23 | 6.60 | 15.06 | -2.76 | 0.04645 | * |
| (VIS + UVA):*Phomopsis* - mock = 0 | -26.70 | 8.76 | 14.05 | -3.05 | 0.04578 | * |
| (VIS + UVA):*Sporobolomyces* - mock = 0 | -17.41 | 7.15 | 16.14 | -2.44 | 0.07160 |  |
| VIS:*Mucor* - mock = 0 | -9.81 | 6.89 | 11.23 | -1.42 | 0.36277 |  |
| VIS:*Diaphorte* - mock = 0 | -25.17 | 8.63 | 15.42 | -2.92 | 0.04578 | * |
| VIS:*Paraphoma* - mock = 0 | -6.40 | 6.52 | 10.59 | -0.98 | 0.55709 |  |
| VIS:*Phomopsis* - mock = 0 | -21.56 | 7.46 | 14.64 | -2.89 | 0.04578 | * |
| VIS:*Sporobolomyces* - mock = 0 | -9.42 | 8.30 | 15.25 | -1.13 | 0.48744 |  |
| mock:VIS – (VIS + UVA) = 0 | -4.12 | 7.72 | 15.49 | -0.53 | 0.67191 |  |
| *Mucor*:VIS - (VIS + UVA) = 0 | -6.38 | 8.23 | 10.64 | -0.78 | 0.60651 |  |
| *Diaphorte*:VIS - (VIS + UVA) = 0 | 3.73 | 7.18 | 11.33 | 0.52 | 0.67191 |  |
| *Paraphoma*:VIS - (VIS + UVA) = 0 | 7.70 | 5.27 | 12.82 | 1.46 | 0.36277 |  |
| *Phomopsis*:VIS - (VIS + UVA) = 0 | 1.02 | 8.60 | 11.85 | 0.12 | 0.90735 |  |
| *Sporobolomyces*:VIS - (VIS + UVA) = 0 | 3.87 | 7.86 | 14.96 | 0.49 | 0.67191 |  |

**B**. Analysis of variance of the root system volume

|  | Num df | Den df | *F* | *p* |  |
| --- | --- | --- | --- | --- | --- |
| endophyte | 5 | 90 | 6.056 | 7.1·10^-5^ | *** |
| light | 1 | 90 | 0.048 | 0.8266 |  |
| endophyte:light | 5 | 90 | 0.790 | 0.5597 |  |

Tests of differences in the mean root system volume

| Hypothesis | Estimate [mm^3^] | SE [mm^3^] | df | *t* | *p_adjusted_* |  |
| --- | --- | --- | --- | --- | --- | --- |
| (VIS + UVA):*Mucor* - mock = 0 | -1.568 | 2.7 | 14.17 | -0.590 | 0.695 |  |
| (VIS + UVA):*Diaphorte* - mock = 0 | -8.791 | 1.9 | 10.55 | -4.650 | 0.013 | * |
| (VIS + UVA):*Paraphoma* - mock = 0 | -4.457 | 2.2 | 12.81 | -2.032 | 0.203 |  |
| (VIS + UVA):*Phomopsis* - mock = 0 | -8.152 | 2.8 | 14.02 | -2.906 | 0.092 |  |
| (VIS + UVA):*Sporobolomyces* - mock | -3.818 | 2.4 | 15.64 | -1.571 | 0.363 |  |
| VIS:*Mucor* - mock = 0 | -2.417 | 2.3 | 11.62 | -1.051 | 0.559 |  |
| VIS:*Diaphorte* - mock = 0 | -5.722 | 2.8 | 15.64 | -2.028 | 0.203 |  |
| VIS:*Paraphoma* - mock = 0 | -0.333 | 2.2 | 10.90 | -0.151 | 0.977 |  |
| VIS:*Phomopsis* - mock = 0 | -6.417 | 2.6 | 15.14 | -2.482 | 0.135 |  |
| VIS:*Sporobolomyces* - mock = 0 | -0.306 | 2.9 | 15.61 | -0.107 | 0.977 |  |
| mock:VIS - (VIS + UVA) = 0 | -1.705 | 2.6 | 13.72 | -0.661 | 0.695 |  |
| *Mucor*:VIS - (VIS + UVA) = 0 | -2.553 | 2.4 | 11.88 | -1.057 | 0.559 |  |
| *Diaphorte*:VIS - (VIS + UVA) = 0 | 1.364 | 2.2 | 10.73 | 0.609 | 0.695 |  |
| *Paraphoma*:VIS - (VIS + UVA) = 0 | 2.420 | 1.8 | 13.78 | 1.356 | 0.450 |  |
| *Phomopsis*:VIS - (VIS + UVA) = 0 | 0.031 | 2.8 | 12.88 | 0.011 | 0.992 |  |
| *Sporobolomyces*:VIS - (VIS + UVA) = 0 | 1.808 | 2.8 | 14.98 | 0.657 | 0.695 |  |

**C**. Analysis of variance of the average root diameter

|  | Num df | Den df | *F* | *p* |  |
| --- | --- | --- | --- | --- | --- |
| endophyte | 5 | 90 | 18.51 | 1.3·10^-12^ | *** |
| light | 1 | 90 | 1.83 | 0.1794 |  |
| endophyte:light | 5 | 90 | 0.87 | 0.5049 |  |

Tests of differences in the mean values of average diameter of roots

| Hypothesis | Estimate  [mm] | SE [mm] | df | *t* | *p_adjusted_* |  |
| --- | --- | --- | --- | --- | --- | --- |
| (VIS + UVA):*Mucor* - mock = 0 | 0.00528 | 0.0030 | 11 | 1.75 | 0.2483 |  |
| (VIS + UVA):*Diaphorte* - mock = 0 | 0.01025 | 0.0025 | 11 | 4.18 | 0.0063 | ** |
| (VIS + UVA):*Paraphoma* - mock = 0 | 0.00740 | 0.0018 | 11 | 4.17 | 0.0063 | ** |
| (VIS + UVA):*Phomopsis* - mock = 0 | 0.00190 | 0.0025 | 11 | 0.76 | 0.5680 |  |
| (VIS + UVA):*Sporobolomyces* - mock = 0 | 0.00856 | 0.0022 | 11 | 3.95 | 0.0072 | ** |
| VIS:*Mucor* - mock = 0 | 0.00232 | 0.0022 | 11 | 1.04 | 0.4583 |  |
| VIS:*Diaphorte* - mock = 0 | 0.01156 | 0.0016 | 11 | 7.02 | 0.0004 | *** |
| VIS:*Paraphoma* - mock = 0 | 0.00680 | 0.0018 | 11 | 3.71 | 0.0092 | ** |
| VIS:*Phomopsis* - mock = 0 | 0.00040 | 0.0021 | 11 | 0.19 | 0.9130 |  |
| VIS:*Sporobolomyces* - mock = 0 | 0.01150 | 0.0019 | 11 | 6.21 | 0.0005 | *** |
| mock:VIS - (VIS + UVA) = 0 | -0.00109 | 0.0022 | 11 | -0.51 | 0.7115 |  |
| *Mucor*:VIS - (VIS + UVA) = 0 | -0.00406 | 0.0031 | 11 | -1.32 | 0.4263 |  |
| *Diaphorte*:VIS - (VIS + UVA) = 0 | 0.00021 | 0.0020 | 11 | 0.10 | 0.9193 |  |
| *Paraphoma*:VIS - (VIS + UVA) = 0 | -0.00169 | 0.0014 | 11 | -1.24 | 0.4312 |  |
| *Phomopsis*:VIS - (VIS + UVA) = 0 | -0.00259 | 0.0025 | 11 | -1.05 | 0.4583 |  |
| *Sporobolomyces*:VIS - (VIS + UVA) = 0 | 0.00185 | 0.0019 | 11 | 0.99 | 0.4583 |  |

**D**. Analysis of variance of the fresh weight of roots

|  | Num df | Den df | *F* | *p* |  |
| --- | --- | --- | --- | --- | --- |
| endophyte | 5 | 91.00 | 2.80 | 0.0211 | * |
| light | 1 | 91.00 | 2.15 | 0.1461 |  |
| endophyte:light | 5 | 91.00 | 1.12 | 0.3545 |  |

Tests of differences in the mean values of the fresh weight of roots

| Hypothesis | Estimate [mg] | SE [mg] | df | *t* | *p_adjusted_* |  |
| --- | --- | --- | --- | --- | --- | --- |
| (VIS + UVA):*Mucor* - mock = 0 | -1.042 | 1.27 | 12.36 | -0.818 | 0.57 |  |
| (VIS + UVA):*Diaphorte* - mock = 0 | -3.355 | 1.11 | 8.39 | -3.010 | 0.15 |  |
| (VIS + UVA):*Paraphoma* - mock = 0 | -3.219 | 1.02 | 6.39 | -3.154 | 0.15 |  |
| (VIS + UVA):*Phomopsis* - mock = 0 | -3.134 | 1.36 | 14.09 | -2.303 | 0.15 |  |
| (VIS + UVA):*Sporobolomyces* - mock = 0 | -2.010 | 0.99 | 5.59 | -2.035 | 0.24 |  |
| VIS:*Mucor* - mock = 0 | -1.872 | 1.29 | 9.48 | -1.448 | 0.32 |  |
| VIS:*Diaphorte* - mock = 0 | -1.756 | 1.56 | 14.95 | -1.123 | 0.44 |  |
| VIS:*Paraphoma* - mock = 0 | -1.467 | 1.37 | 11.22 | -1.074 | 0.44 |  |
| VIS:*Phomopsis* - mock = 0 | -3.126 | 1.63 | 15.46 | -1.917 | 0.24 |  |
| VIS:*Sporobolomyces* - mock = 0 | -0.727 | 1.42 | 12.77 | -0.512 | 0.71 |  |
| mock:VIS - (VIS + UVA) = 0 | 0.083 | 1.53 | 11.78 | 0.054 | 0.96 |  |
| *Mucor*:VIS - (VIS + UVA) = 0 | -0.747 | 0.99 | 11.98 | -0.752 | 0.57 |  |
| *Diaphorte*:VIS - (VIS + UVA) = 0 | 1.683 | 1.17 | 13.88 | 1.433 | 0.32 |  |
| *Paraphoma*:VIS - (VIS + UVA) = 0 | 1.834 | 0.78 | 13.79 | 2.366 | 0.15 |  |
| *Phomopsis*:VIS - (VIS + UVA) = 0 | 0.091 | 1.48 | 14.44 | 0.061 | 0.96 |  |
| *Sporobolomyces*:VIS - (VIS + UVA) = 0 | 1.366 | 0.83 | 10.62 | 1.649 | 0.29 |  |

**E**. Analysis of variance of the fresh weight of shoots

|  | Num df | Den df | *F* | *p* |  |
| --- | --- | --- | --- | --- | --- |
| endophyte | 5 | 91 | 9.82 | 1.6·10^-7^ | *** |
| light | 1 | 91 | 81.93 | 2.5·10^-14^ | *** |
| endophyte:light | 5 | 91 | 0.53 | 0.7536 |  |

Tests of differences in the mean values of the fresh weight of shoots

| Hypothesis | Estimate [mg] | SE [mg] | df | *t* | *p_adjusted_* |  |
| --- | --- | --- | --- | --- | --- | --- |
| (VIS + UVA):*Mucor* - mock = 0 | -2.66 | 0.89 | 10.48 | -2.98 | 0.02276 | * |
| (VIS + UVA):*Diaphorte* - mock = 0 | -1.63 | 0.74 | 12.79 | -2.20 | 0.06202 |  |
| (VIS + UVA):*Paraphoma* - mock = 0 | -4.96 | 0.70 | 10.62 | -7.13 | 0.00037 | *** |
| (VIS + UVA):*Phomopsis* - mock = 0 | -0.56 | 1.06 | 9.16 | -0.53 | 0.65088 |  |
| (VIS + UVA):*Sporobolomyces* - mock = 0 | -4.44 | 0.81 | 10.94 | -5.48 | 0.00104 | ** |
| VIS:*Mucor* - mock = 0 | -3.12 | 1.24 | 11.41 | -2.51 | 0.04115 | * |
| VIS:*Diaphorte* - mock = 0 | -1.20 | 1.59 | 11.44 | -0.75 | 0.53346 |  |
| VIS:*Paraphoma* - mock = 0 | -3.87 | 1.35 | 11.78 | -2.87 | 0.02276 | * |
| VIS:*Phomopsis* - mock = 0 | 0.50 | 1.68 | 11.65 | 0.30 | 0.77208 |  |
| VIS:*Sporobolomyces* - mock = 0 | -2.60 | 1.26 | 11.23 | -2.07 | 0.07701 |  |
| mock:VIS - (VIS + UVA) = 0 | 4.33 | 0.97 | 8.85 | 4.45 | 0.00537 | ** |
| *Mucor*:VIS - (VIS + UVA) = 0 | 3.86 | 1.20 | 14.02 | 3.22 | 0.01628 | * |
| *Diaphorte*:VIS - (VIS + UVA) = 0 | 4.76 | 1.47 | 10.83 | 3.24 | 0.01724 | * |
| *Paraphoma*:VIS - (VIS + UVA) = 0 | 5.42 | 1.18 | 11.61 | 4.59 | 0.00270 | ** |
| *Phomopsis*:VIS - (VIS + UVA) = 0 | 5.39 | 1.75 | 13.21 | 3.08 | 0.01724 | * |
| *Sporobolomyces*:VIS - (VIS + UVA) = 0 | 6.18 | 1.15 | 13.26 | 5.37 | 0.00096 | *** |

**F**. Analysis of variance of the shoot anthocyanin content

|  | Num df | Den df | *F* | *p* |  |
| --- | --- | --- | --- | --- | --- |
| endophyte | 5 | 82 | 2.24 | 0.0576 |  |
| light | 1 | 82 | 22.40 | 9.1·10^-6^ | *** |
| endophyte:light | 5 | 82 | 1.11 | 0.3603 |  |

Tests of differences in the mean values of the shoot anthocyanin content

| Hypothesis | Estimate | SE | df | *t* | *p_adjusted_* |  |
| --- | --- | --- | --- | --- | --- | --- |
| (VIS + UVA):*Mucor* - mock = 0 | -0.066 | 0.47 | 12.59 | -0.141 | 0.95 |  |
| (VIS + UVA):*Diaphorte* - mock = 0 | 0.471 | 0.70 | 10.84 | 0.668 | 0.76 |  |
| (VIS + UVA):*Paraphoma* - mock = 0 | 2.325 | 0.97 | 6.37 | 2.391 | 0.28 |  |
| (VIS + UVA):*Phomopsis* - mock = 0 | -0.069 | 0.65 | 13.39 | -0.105 | 0.95 |  |
| (VIS + UVA):*Sporobolomyces* - mock = 0 | 1.669 | 1.24 | 6.98 | 1.347 | 0.44 |  |
| VIS:*Mucor* - mock = 0 | -0.333 | 0.34 | 10.78 | -0.992 | 0.61 |  |
| VIS:*Diaphorte* - mock = 0 | 0.026 | 0.42 | 15.09 | 0.064 | 0.95 |  |
| VIS:*Paraphoma* - mock = 0 | 0.222 | 0.36 | 14.37 | 0.620 | 0.76 |  |
| VIS:*Phomopsis* - mock = 0 | -0.232 | 0.40 | 14.34 | -0.577 | 0.76 |  |
| VIS:*Sporobolomyces* - mock = 0 | -0.043 | 0.36 | 12.40 | -0.118 | 0.95 |  |
| mock:VIS - (VIS + UVA) = 0 | -0.711 | 0.45 | 14.62 | -1.571 | 0.37 |  |
| *Mucor*:VIS - (VIS + UVA) = 0 | -0.979 | 0.36 | 10.30 | -2.732 | 0.21 |  |
| *Diaphorte*:VIS - (VIS + UVA) = 0 | -1.156 | 0.68 | 10.34 | -1.690 | 0.37 |  |
| *Paraphoma*:VIS - (VIS + UVA) = 0 | -2.814 | 0.93 | 5.53 | -3.013 | 0.21 |  |
| *Phomopsis*:VIS - (VIS + UVA) = 0 | -0.874 | 0.62 | 12.26 | -1.403 | 0.42 |  |
| *Sporobolomyces*:VIS - (VIS + UVA) = 0 | -2.423 | 1.21 | 6.32 | -2.002 | 0.36 |  |

Table S3. Results of the statistical analysis of the effects of endophyte inoculation (six levels, including the mock-inoculated control) and light conditions (two levels: only visible light /VIS/, visible light supplemented with UV-A /VIS + UVA/) on the log-transformed relative level of the following transcripts in Arabidopsis plants: *CHS1* (A), *PAL1* (B), *ICS1* (C), *PDF1.2* (D), *CRY1* (E), *CRY2* (F), *PHOT1* (G), *PHOT2* (H), *UVR8* (I). A linear model of the relationship between log-transformed transcript levels and predictors (endophyte, light conditions and their interaction) was fitted using the *gls* command of the *nlme* package in the R software. To control for heteroskedasticity, the model allowed for different variance in each group (*nlme* variance function *varIdent(form = ~ 1| light*endophyte)*). The type III analysis of variance was performed using the *anova* command, with the effects coding of factor levels. Differences between cell means of log-transformed transcript levels were examined using the *emmeans* package, with the approximate number of degrees of freedom calculated using Satterthwaite’s method. *p* values are adjusted for the false discovery rate of 0.05 using the Benjamin-Hochberg method.

**A**. Analysis of variance of the log-transformed *CHS1* transcript levels

| Denominator df: 91 | | | | |
| --- | --- | --- | --- | --- |
|  | Num df. | *F* | *p* |  |
| endophyte | 5 | 3.280821 | 0.0089 | ** |
| light | 1 | 3.426037 | 0.0673 |  |
| endophyte:light | 5 | 0.068470 | 0.9967 |  |

Tests of differences in the mean log-transformed *CHS1* transcript levels

| Hypothesis | Estimate | SE | df | *t* | *p_adjusted_* |  |
| --- | --- | --- | --- | --- | --- | --- |
| (VIS + UVA):*Mucor* - mock = 0 | -0.3907 | 0.39 | 16.21 | -0.999 | 0.71 |  |
| (VIS + UVA):*Diaphorte* - mock = 0 | -0.3487 | 0.41 | 14.91 | -0.842 | 0.71 |  |
| (VIS + UVA):*Paraphoma* - mock = 0 | 0.3688 | 0.35 | 13.64 | 1.044 | 0.71 |  |
| (VIS + UVA):*Phomopsis* - mock = 0 | -0.0062 | 0.35 | 14.27 | -0.018 | 0.99 |  |
| (VIS + UVA):*Sporobolomyces* - mock = 0 | -0.5756 | 0.41 | 14.69 | -1.414 | 0.71 |  |
| VIS:*Mucor* - mock = 0 | -0.3116 | 0.47 | 14.97 | -0.658 | 0.71 |  |
| VIS:*Diaphorte* - mock = 0 | -0.4351 | 0.48 | 15.16 | -0.914 | 0.71 |  |
| VIS:*Paraphoma* - mock = 0 | 0.2843 | 0.47 | 14.89 | 0.605 | 0.71 |  |
| VIS:*Phomopsis* - mock = 0 | 0.1605 | 0.51 | 15.88 | 0.314 | 0.81 |  |
| VIS:*Sporobolomyces* - mock = 0 | -0.6627 | 0.45 | 13.96 | -1.476 | 0.71 |  |
| mock:VIS - (VIS + UVA) = 0 | -0.3097 | 0.46 | 14.79 | -0.672 | 0.71 |  |
| *Mucor*:VIS - (VIS + UVA) = 0 | -0.2306 | 0.41 | 15.33 | -0.568 | 0.71 |  |
| *Diaphorte*:VIS - (VIS + UVA) = 0 | -0.3962 | 0.43 | 16.04 | -0.920 | 0.71 |  |
| *Paraphoma*:VIS - (VIS + UVA) = 0 | -0.3943 | 0.36 | 15.16 | -1.082 | 0.71 |  |
| *Phomopsis*:VIS - (VIS + UVA) = 0 | -0.1431 | 0.41 | 13.37 | -0.346 | 0.81 |  |
| *Sporobolomyces*:VIS - (VIS + UVA) = 0 | -0.3968 | 0.39 | 15.55 | -1.009 | 0.71 |  |

**B.** Analysis of variance of the log-transformed *PAL1* transcript levels

| Denominator df: 96 | | | | |
| --- | --- | --- | --- | --- |
|  | Num. df | *F* | *p* |  |
| endophyte | 5 | 4.31824 | 0.0014 | ** |
| light | 1 | 5.74971 | 0.0184 | * |
| endophyte:light | 5 | 0.37840 | 0.8624 |  |

Tests of differences in the mean log-transformed *PAL1* transcript levels

| Hypothesis | Estimate | SE | df | *t* | *p_adjusted_* |  |
| --- | --- | --- | --- | --- | --- | --- |
| (VIS + UVA):*Mucor* - mock = 0 | -0.147 | 0.24 | 12.19 | -0.60 | 0.80 |  |
| (VIS + UVA):*Diaphorte* - mock = 0 | 0.179 | 0.21 | 9.83 | 0.85 | 0.80 |  |
| (VIS + UVA):*Paraphoma* - mock = 0 | 0.443 | 0.21 | 12.08 | 2.10 | 0.29 |  |
| (VIS + UVA):*Phomopsis* - mock = 0 | 0.381 | 0.19 | 9.52 | 2.02 | 0.29 |  |
| (VIS + UVA):*Sporobolomyces* - mock = 0 | 0.048 | 0.21 | 10.79 | 0.23 | 0.88 |  |
| VIS:*Mucor* - mock = 0 | -0.224 | 0.35 | 13.38 | -0.64 | 0.80 |  |
| VIS:*Diaphorte* - mock = 0 | -0.174 | 0.33 | 11.69 | -0.52 | 0.80 |  |
| VIS:*Paraphoma* - mock = 0 | 0.336 | 0.33 | 11.70 | 1.00 | 0.80 |  |
| VIS:*Phomopsis* - mock = 0 | 0.132 | 0.33 | 11.62 | 0.40 | 0.80 |  |
| VIS:*Sporobolomyces* - mock = 0 | -0.305 | 0.33 | 11.26 | -0.93 | 0.80 |  |
| mock:VIS - (VIS + UVA) = 0 | -0.044 | 0.34 | 11.26 | -0.13 | 0.90 |  |
| *Mucor*:VIS - (VIS + UVA) = 0 | -0.122 | 0.27 | 15.98 | -0.46 | 0.80 |  |
| *Diaphorte*:VIS - (VIS + UVA) = 0 | -0.397 | 0.21 | 16.68 | -1.91 | 0.29 |  |
| *Paraphoma*:VIS - (VIS + UVA) = 0 | -0.151 | 0.21 | 15.58 | -0.73 | 0.80 |  |
| *Phomopsis*:VIS - (VIS + UVA) = 0 | -0.293 | 0.19 | 14.32 | -1.58 | 0.43 |  |
| *Sporobolomyces*:VIS - (VIS + UVA) = 0 | -0.398 | 0.20 | 16.15 | -2.00 | 0.29 |  |

**C.** Analysis of variance of the log-transformed *ICS1* transcript levels

| Denominator df: 96 | | | | |
| --- | --- | --- | --- | --- |
|  | Num. df | *F* | *p* |  |
| endophyte | 5 | 11.5419310 | 9.7 ·10^-9^ | *** |
| light | 1 | 9.4750342 | 0.0027 | ** |
| endophyte:light | 5 | 0.7119521 | 0.6159 |  |

Tests of differences in the mean log-transformed *ICS1* transcript levels

| Hypothesis | Estimate | SE | df | *t* | *p_adjusted_* |  |
| --- | --- | --- | --- | --- | --- | --- |
| (VIS + UVA):*Mucor* - mock = 0 | 0.211 | 0.19 | 9.47 | 1.107 | 0.3377 |  |
| (VIS + UVA):*Diaphorte* - mock = 0 | 0.336 | 0.18 | 9.75 | 1.917 | 0.1405 |  |
| (VIS + UVA):*Paraphoma* - mock = 0 | 0.570 | 0.12 | 11.98 | 4.717 | 0.0034 | ** |
| (VIS + UVA):*Phomopsis* - mock = 0 | 0.474 | 0.18 | 9.68 | 2.657 | 0.0789 |  |
| (VIS + UVA):*Sporobolomyces* - mock = 0 | 0.912 | 0.21 | 9.21 | 4.379 | 0.0067 | ** |
| VIS:*Mucor* - mock = 0 | 0.348 | 0.20 | 15.61 | 1.746 | 0.1461 |  |
| VIS:*Diaphorte* - mock = 0 | 0.364 | 0.15 | 13.30 | 2.397 | 0.0850 |  |
| VIS:*Paraphoma* - mock = 0 | 0.860 | 0.20 | 15.51 | 4.264 | 0.0034 | ** |
| VIS:*Phomopsis* - mock = 0 | 0.306 | 0.17 | 15.46 | 1.822 | 0.1405 |  |
| VIS:*Sporobolomyces* - mock = 0 | 0.788 | 0.18 | 16.00 | 4.291 | 0.0034 | ** |
| mock:VIS - (VIS + UVA) = 0 | -0.280 | 0.14 | 10.84 | -1.990 | 0.1405 |  |
| *Mucor*:VIS - (VIS + UVA) = 0 | -0.143 | 0.24 | 15.50 | -0.602 | 0.5928 |  |
| *Diaphorte*:VIS - (VIS + UVA) = 0 | -0.252 | 0.18 | 11.48 | -1.366 | 0.2438 |  |
| *Paraphoma*:VIS - (VIS + UVA) = 0 | 0.011 | 0.19 | 14.25 | 0.056 | 0.9563 |  |
| *Phomopsis*:VIS - (VIS + UVA) = 0 | -0.449 | 0.20 | 13.49 | -2.235 | 0.0980 |  |
| *Sporobolomyces*:VIS - (VIS + UVA) = 0 | -0.405 | 0.24 | 13.72 | -1.690 | 0.1514 |  |

**D.** Analysis of variance of the log-transformed *PDF1.2* transcript levels

| Denominator df: 96 | | | | |
| --- | --- | --- | --- | --- |
|  | Num df. | *F* | *p* |  |
| endophyte | 5 | 12.0641 | 4.5 ·10^-9^ | *** |
| light | 1 | 2.1078 | 0.1498 |  |
| endophyte:light | 5 | 2.9283 | 0.0167 | * |

Tests of differences in the mean log-transformed *PDF1.2* transcript levels

| Hypothesis | Estimate | SE | df | *t* | *p_adjusted_* |  |
| --- | --- | --- | --- | --- | --- | --- |
| (VIS + UVA):*Mucor* - mock = 0 | 1.72 | 0.66 | 12.71 | 2.60 | 0.0715 |  |
| (VIS + UVA):*Diaphorte* - mock = 0 | 1.80 | 0.55 | 14.65 | 3.25 | 0.0293 | * |
| (VIS + UVA):*Paraphoma* - mock = 0 | 3.61 | 0.47 | 15.17 | 7.60 | 2.4 ·10^-5^ | *** |
| (VIS + UVA):*Phomopsis* - mock = 0 | 1.45 | 0.76 | 11.48 | 1.91 | 0.1848 |  |
| (VIS + UVA):*Sporobolomyces* - mock = 0 | 3.41 | 0.53 | 14.89 | 6.38 | 0.0001 | *** |
| VIS:*Mucor* - mock = 0s | -0.96 | 0.59 | 11.34 | -1.63 | 0.1902 |  |
| VIS:*Diaphorte* - mock = 0 | -0.23 | 0.69 | 15.55 | -0.34 | 0.7913 |  |
| VIS:*Paraphoma* - mock = 0 | 0.86 | 0.61 | 12.74 | 1.40 | 0.2484 |  |
| VIS:*Phomopsis* - mock = 0 | -0.25 | 0.73 | 15.96 | -0.34 | 0.7913 |  |
| VIS:*Sporobolomyces* - mock = 0 | 1.02 | 0.62 | 13.08 | 1.64 | 0.1902 |  |
| control:VIS - (VIS + UVA) = 0 | 1.55 | 0.63 | 13.16 | 2.47 | 0.0739 |  |
| *Mucor*:VIS - (VIS + UVA) = 0 | -1.13 | 0.62 | 10.92 | -1.81 | 0.1902 |  |
| *Diaphorte*:VIS - (VIS + UVA) = 0 | -0.48 | 0.63 | 15.99 | -0.76 | 0.5629 |  |
| *Paraphoma*:VIS - (VIS + UVA) = 0 | -1.20 | 0.45 | 15.55 | -2.64 | 0.0715 |  |
| *Phomopsis*:VIS - (VIS + UVA) = 0 | -0.15 | 0.84 | 14.67 | -0.18 | 0.8609 |  |
| *Sporobolomyces*:VIS - (VIS + UVA) = 0 | -0.84 | 0.52 | 14.76 | -1.60 | 0.1902 |  |

**E.** Analysis of variance of the log-transformed *CRY1* transcript levels

| Denominator df: 42 | | | | |
| --- | --- | --- | --- | --- |
|  | Num. df | *F* | *p* |  |
| endophyte | 5 | 2.2136 | 0.071 |  |
| light | 1 | 0.0413 | 0.840 |  |
| endophyte:light | 5 | 1.8007 | 0.134 |  |

Tests of differences in the mean log-transformed *CRY1* transcript levels

| Hypothesis | Estimate | SE | df | *t* | *p_adjusted_* |  |
| --- | --- | --- | --- | --- | --- | --- |
| (VIS + UVA):*Mucor* - mock = 0 | 0.247 | 0.24 | 4.37 | 1.041 | 0.65 |  |
| (VIS + UVA):*Diaphorte* - mock = 0 | 0.079 | 0.31 | 5.84 | 0.257 | 0.94 |  |
| (VIS + UVA):*Paraphoma* - mock = 0 | -0.422 | 0.23 | 4.09 | -1.811 | 0.65 |  |
| (VIS + UVA):*Phomopsis* - mock = 0 | 0.143 | 0.26 | 5.47 | 0.546 | 0.88 |  |
| (VIS + UVA):*Sporobolomyces* - mock = 0 | -0.039 | 0.26 | 5.27 | -0.151 | 0.94 |  |
| VIS:*Mucor* - mock = 0 | 0.859 | 0.67 | 4.64 | 1.273 | 0.65 |  |
| VIS:*Diaphorte* - mock = 0 | 0.921 | 0.66 | 4.33 | 1.390 | 0.65 |  |
| VIS:*Paraphoma* - mock = 0 | 0.848 | 0.67 | 4.45 | 1.272 | 0.65 |  |
| VIS:*Phomopsis* - mock = 0 | 0.911 | 0.66 | 4.19 | 1.388 | 0.65 |  |
| VIS:*Sporobolomyces* - mock = 0 | 0.679 | 0.67 | 4.62 | 1.008 | 0.65 |  |
| mock:VIS - (VIS + UVA) = 0 | -0.730 | 0.68 | 4.86 | -1.067 | 0.65 |  |
| *Mucor*:VIS - (VIS + UVA) = 0 | -0.118 | 0.21 | 6.09 | -0.565 | 0.88 |  |
| *Diaphorte*:VIS - (VIS + UVA) = 0 | 0.112 | 0.26 | 4.92 | 0.432 | 0.91 |  |
| *Paraphoma*:VIS - (VIS + UVA) = 0 | 0.540 | 0.18 | 6.12 | 3.051 | 0.35 |  |
| *Phomopsis*:VIS - (VIS + UVA) = 0 | 0.039 | 0.18 | 5.48 | 0.218 | 0.94 |  |
| *Sporobolomyces*:VIS - (VIS + UVA) = 0 | -0.012 | 0.23 | 6.99 | -0.051 | 0.96 |  |

**F.** Analysis of variance of the log-transformed *CRY2* transcript levels

| Denominator df: 42 | | | | |
| --- | --- | --- | --- | --- |
|  | Num. df | *F* | *p* |  |
| endophyte | 5 | 0.17 | 0.97 |  |
| light | 1 | 1.05 | 0.31 |  |
| endophyte:light | 5 | 0.64 | 0.67 |  |

| Hypothesis | Estimate | SE | df | *t* | *p_adjusted_* |  |
| --- | --- | --- | --- | --- | --- | --- |
| (VIS + UVA):*Mucor* - mock = 0 | 0.247 | 0.24 | 4.37 | 1.041 | 0.65 |  |
| (VIS + UVA):*Diaphorte* - mock = 0 | 0.079 | 0.31 | 5.84 | 0.257 | 0.94 |  |
| (VIS + UVA):*Paraphoma* - mock = 0 | -0.422 | 0.23 | 4.09 | -1.811 | 0.65 |  |
| (VIS + UVA):*Phomopsis* - mock = 0 | 0.143 | 0.26 | 5.47 | 0.546 | 0.88 |  |
| (VIS + UVA):*Sporobolomyces* - mock = 0 | -0.039 | 0.26 | 5.27 | -0.151 | 0.94 |  |
| VIS:*Mucor* - mock = 0 | 0.859 | 0.67 | 4.64 | 1.273 | 0.65 |  |
| VIS:*Diaphorte* - mock = 0 | 0.921 | 0.66 | 4.33 | 1.390 | 0.65 |  |
| VIS:*Paraphoma* - mock = 0 | 0.848 | 0.67 | 4.45 | 1.272 | 0.65 |  |
| VIS:*Phomopsis* - mock = 0 | 0.911 | 0.66 | 4.19 | 1.388 | 0.65 |  |
| VIS:*Sporobolomyces* - mock = 0 | 0.679 | 0.67 | 4.62 | 1.008 | 0.65 |  |
| mock:VIS - (VIS + UVA) = 0 | -0.730 | 0.68 | 4.86 | -1.067 | 0.65 |  |
| *Mucor*:VIS - (VIS + UVA) = 0 | -0.118 | 0.21 | 6.09 | -0.565 | 0.88 |  |
| *Diaphorte*:VIS - (VIS + UVA) = 0 | 0.112 | 0.26 | 4.92 | 0.432 | 0.91 |  |
| *Paraphoma*:VIS - (VIS + UVA) = 0 | 0.540 | 0.18 | 6.12 | 3.051 | 0.35 |  |
| *Phomopsis*:VIS - (VIS + UVA) = 0 | 0.039 | 0.18 | 5.48 | 0.218 | 0.94 |  |
| *Sporobolomyces*:VIS - (VIS + UVA) = 0 | -0.012 | 0.23 | 6.99 | -0.051 | 0.96 |  |

**G.** Analysis of variance of the log-transformed *PHOT1* transcript levels

| Denominator df: 96 | | | | |
| --- | --- | --- | --- | --- |
|  | Num df. | *F* | *p* |  |
| endophyte | 5 | 0.6598 | 0.655 |  |
| light | 1 | 4.7042 | 0.033 | * |
| endophyte:light | 5 | 0.6279 | 0.679 |  |

Tests of differences in the mean log-transformed *PHOT1* transcript levels

| Hypothesis | Estimate | SE | df | *t* | *p_adjusted_* |  |
| --- | --- | --- | --- | --- | --- | --- |
| (VIS + UVA):*Mucor* - mock = 0 | 0.020 | 0.14 | 14.82 | 0.14 | 0.89 |  |
| (VIS + UVA):*Diaphorte* - mock = 0 | 0.035 | 0.16 | 15.95 | 0.21 | 0.89 |  |
| (VIS + UVA):*Paraphoma* - mock = 0 | -0.251 | 0.13 | 12.55 | -1.93 | 0.61 |  |
| (VIS + UVA):*Phomopsis* - mock = 0 | 0.031 | 0.20 | 14.19 | 0.16 | 0.89 |  |
| (VIS + UVA):*Sporobolomyces* - mock = 0 | -0.112 | 0.16 | 16.24 | -0.69 | 0.89 |  |
| VIS:*Mucor* - mock = 0 | 0.121 | 0.23 | 15.05 | 0.52 | 0.89 |  |
| VIS:*Diaphorte* - mock = 0 | 0.141 | 0.19 | 15.94 | 0.73 | 0.89 |  |
| VIS:*Paraphoma* - mock = 0 | 0.104 | 0.21 | 15.94 | 0.51 | 0.89 |  |
| VIS:*Phomopsis* - mock = 0 | 0.031 | 0.17 | 14.67 | 0.18 | 0.89 |  |
| VIS:*Sporobolomyces* - mock = 0 | -0.093 | 0.22 | 15.47 | -0.42 | 0.89 |  |
| mock:VIS - (VIS + UVA) = 0 | 0.070 | 0.18 | 14.89 | 0.39 | 0.89 |  |
| *Mucor*:VIS - (VIS + UVA) = 0 | 0.171 | 0.20 | 11.90 | 0.84 | 0.89 |  |
| *Diaphorte*:VIS - (VIS + UVA) = 0 | 0.176 | 0.18 | 15.83 | 0.97 | 0.89 |  |
| *Paraphoma*:VIS - (VIS + UVA) = 0 | 0.425 | 0.16 | 11.15 | 2.59 | 0.40 |  |
| *Phomopsis*:VIS - (VIS + UVA) = 0 | 0.069 | 0.19 | 13.61 | 0.36 | 0.89 |  |
| *Sporobolomyces*:VIS - (VIS + UVA) = 0 | 0.089 | 0.21 | 14.35 | 0.43 | 0.89 |  |

**H.** Analysis of variance of the log-transformed *PHOT2* transcript levels

| Denominator df: 96 | | | | |
| --- | --- | --- | --- | --- |
|  | Num. df | *F* | *p* |  |
| endophyte | 5 | 2.834 | 0.020 | * |
| light | 1 | 4.484 | 0.037 | * |
| endophyte:light | 5 | 1.036 | 0.401 |  |

Tests of differences in the mean log-transformed *PHOT2* transcript levels

| Hypothesis | Estimate | SE | df | *t* | *p_adjusted_* |  |
| --- | --- | --- | --- | --- | --- | --- |
| (VIS + UVA):*Mucor* - mock = 0 | -0.1254 | 0.17 | 16.05 | -0.751 | 0.82 |  |
| (VIS + UVA):*Diaphorte* - mock = 0 | -0.1029 | 0.23 | 13.67 | -0.454 | 0.88 |  |
| (VIS + UVA):*Paraphoma* - mock = 0 | -0.2881 | 0.18 | 16.43 | -1.642 | 0.48 |  |
| (VIS + UVA):*Phomopsis* - mock = 0 | 0.0482 | 0.17 | 15.45 | 0.279 | 0.88 |  |
| (VIS + UVA):*Sporobolomyces* - mock = 0 | -0.3834 | 0.19 | 15.19 | -2.048 | 0.31 |  |
| VIS:*Mucor* - mock = 0 | -0.3311 | 0.24 | 13.00 | -1.360 | 0.63 |  |
| VIS:*Diaphorte* - mock = 0 | -0.1473 | 0.23 | 11.47 | -0.634 | 0.86 |  |
| VIS:*Paraphoma* - mock = 0 | -0.1191 | 0.22 | 10.23 | -0.532 | 0.88 |  |
| VIS:*Phomopsis* - mock = 0 | -0.2161 | 0.24 | 12.68 | -0.894 | 0.78 |  |
| VIS:*Sporobolomyces* - mock = 0 | -0.6002 | 0.25 | 13.56 | -2.415 | 0.24 |  |
| mock:VIS - (VIS + UVA) = 0 | 0.2601 | 0.24 | 12.91 | 1.071 | 0.77 |  |
| *Mucor*:VIS - (VIS + UVA) = 0 | 0.0544 | 0.17 | 16.06 | 0.324 | 0.88 |  |
| *Diaphorte*:VIS - (VIS + UVA) = 0 | 0.2158 | 0.22 | 12.11 | 1.003 | 0.77 |  |
| *Paraphoma*:VIS - (VIS + UVA) = 0 | 0.4292 | 0.15 | 12.66 | 2.900 | 0.20 |  |
| *Phomopsis*:VIS - (VIS + UVA) = 0 | -0.0042 | 0.17 | 15.49 | -0.025 | 0.98 |  |
| *Sporobolomyces*:VIS - (VIS + UVA) = 0 | 0.0433 | 0.19 | 15.78 | 0.223 | 0.88 |  |

**I.** Analysis of variance of the log-transformed *UVR8* transcript levels

| Denominator df: 42 | | | | |
| --- | --- | --- | --- | --- |
|  | Num. df | *F* | *p* |  |
| endophyte | 5 | 0.673 | 0.647 |  |
| light | 1 | 1.689 | 0.201 |  |
| endophyte:light | 5 | 0.305 | 0.907 |  |

Tests of differences in the mean log-transformed *UVR8* transcript levels

| Hypothesis | Estimate | SE | df | *t* | *p_adjusted_* |  |
| --- | --- | --- | --- | --- | --- | --- |
| (VIS + UVA):*Mucor* - mock = 0 | -0.0898 | 0.29 | 6.42 | -0.313 | 0.98 |  |
| (VIS + UVA):*Diaphorte* - mock = 0 | 0.0440 | 0.23 | 6.18 | 0.190 | 0.98 |  |
| (VIS + UVA):Paraphoma - mock = 0 | -0.0065 | 0.27 | 7.27 | -0.024 | 0.98 |  |
| (VIS + UVA):*Phomopsis* - mock = 0 | -0.0929 | 0.33 | 5.77 | -0.282 | 0.98 |  |
| (VIS + UVA):*Sporobolomyces* - mock = 0 | -0.0176 | 0.23 | 6.28 | -0.076 | 0.98 |  |
| VIS:*Mucor* - mock = 0 | 1.4352 | 1.51 | 4.05 | 0.948 | 0.98 |  |
| VIS:*Diaphorte* - mock = 0 | 1.6527 | 1.52 | 4.08 | 1.090 | 0.98 |  |
| VIS:*Paraphoma* - mock = 0 | 1.4930 | 1.51 | 4.05 | 0.986 | 0.98 |  |
| VIS:*Phomopsis* - mock = 0 | 1.2998 | 1.52 | 4.10 | 0.856 | 0.98 |  |
| VIS:*Sporobolomyces* - mock = 0 | 1.5967 | 1.52 | 4.09 | 1.052 | 0.98 |  |
| mock:VIS - (VIS + UVA) = 0 | -1.6230 | 1.52 | 4.13 | -1.067 | 0.98 |  |
| *Mucor*:VIS - (VIS + UVA) = 0 | -0.0980 | 0.24 | 4.69 | -0.406 | 0.98 |  |
| *Diaphorte*:VIS - (VIS + UVA) = 0 | -0.0143 | 0.20 | 6.93 | -0.072 | 0.98 |  |
| *Paraphoma*:VIS - (VIS + UVA) = 0 | -0.1235 | 0.23 | 5.35 | -0.545 | 0.98 |  |
| *Phomopsis*:VIS - (VIS + UVA) = 0 | -0.2302 | 0.32 | 5.22 | -0.728 | 0.98 |  |
| *Sporobolomyces*:VIS - (VIS + UVA) = 0 | -0.0087 | 0.21 | 6.71 | -0.042 | 0.98 |  |

Table S4. Results of the statistical analysis of the effects of endophyte inoculation (two levels, the mock-inoculated control and inoculation with *Sporobolomyces* *ruberrimus*), light conditions (two levels, only visible light /VIS/ or visible light supplemented with UV-A /VIS + UVA/) and the interaction between endophyte presence and light conditions on the leaf flavonol (A) and chlorophyll (B) content indices measured with Dualex, as well as the lipid peroxidation level (C) examined with the MDA assay. A linear model was fitted using the *gls* command of the *nlme* package in the R software. To control for heteroskedasticity, the model allowed for different variance in each group (*nlme* variance function *varIdent(form = ~ 1| light*endophyte)*). The type III analysis of variance was performed using the *anova* command, with the effects coding of factor levels. Differences between cell means were examined using the *emmeans* package, with the approximate number of degrees of freedom calculated using Satterthwaite’s method. *p* values are adjusted for the false discovery rate of 0.05 using the Benjamin-Hochberg method.

**A**. Analysis of variance of the Dualex flavonol content index

| Denominator df:69 | | | | |
| --- | --- | --- | --- | --- |
|  | Num. df | *F* | *p* |  |
| endophyte | 1 | 2.37 | 0.1282 |  |
| light | 1 | 0.09 | 0.7647 |  |
| endophyte:light | 1 | 6.32 | 0.0143 | * |

Tests of differences in the mean values the Dualex flavonol content index

| Hypothesis | Estimate | SE | df | *t* | *p_adjusted_* |  |
| --- | --- | --- | --- | --- | --- | --- |
| mock:(VIS + UVA)-VIS = 0 | -0.0175 | 0.0103 | 31.02 | -1.69 | 0.1337 |  |
| *Sporobolomyces*:(VIS + UVA)-VIS = 0 | 0.0222 | 0.0120 | 29.30 | 1.86 | 0.1337 |  |
| (VIS + UVA):*Sporobolomyces* - mock = 0 | 0.0320 | 0.0128 | 30.97 | 2.51 | 0.0701 |  |
| VIS:*Sporobolomyces* - mock = 0 | -0.0077 | 0.0093 | 32.60 | -0.83 | 0.4148 |  |

**B**. Analysis of variance of the Dualex chlorophyll content index

| Denominator df:69 | | | | |
| --- | --- | --- | --- | --- |
|  | Num. df | *F* | *p* |  |
| endophyte | 1 | 0.26 | 0.6118 |  |
| light | 1 | 4.24 | 0.0432 | * |
| endophyte:light | 1 | 0.11 | 0.7363 |  |

Tests of differences in the mean values the Dualex chlorophyll content index

| Hypothesis | Estimate | SE | df | *t* | *p_adjusted_* |  |
| --- | --- | --- | --- | --- | --- | --- |
| mock:(VIS + UVA)-VIS = 0 | 1.51 | 1.24 | 29.52 | 1.22 | 0.4655 |  |
| *Sporobolomyces*:(VIS + UVA)-VIS = 0 | 2.11 | 1.24 | 34.49 | 1.70 | 0.3963 |  |
| (VIS + UVA):*Sporobolomyces* - mock = 0 | -0.15 | 1.25 | 32.08 | -0.12 | 0.9045 |  |
| VIS:*Sporobolomyces* - mock = 0 | -0.75 | 1.24 | 31.60 | -0.60 | 0.7358 |  |

**C**. Analysis of variance of the lipid peroxidation levels examined with the MDA assay

| Denominator df:65 | | | | |
| --- | --- | --- | --- | --- |
|  | Num. df | *F* | *p* |  |
| endophyte | 1 | 1.82 | 0.1821 |  |
| light | 1 | 0.12 | 0.7338 |  |
| endophyte:light | 1 | 0.54 | 0.4657 |  |

Tests of differences in the mean values the lipid peroxidation levels

| Hypothesis | Estimate  [mol ·g^-1^] | SE [mol ·g^-1^] | df | *t* | *p_adjusted_* |
| --- | --- | --- | --- | --- | --- |
| mock:(VIS + UVA)-VIS = 0 | -0.46 | 1.77 | 19.69 | -0.26 | 0.7988 |
| *Sporobolomyces*:(VIS + UVA)-VIS = 0 | 1.26 | 1.52 | 34.99 | 0.83 | 0.7988 |
| (VIS + UVA):*Sporobolomyces* - mock = 0 | 2.43 | 1.41 | 34.57 | 1.72 | 0.3773 |
| VIS:*Sporobolomyces* - mock = 0 | 0.72 | 1.86 | 22.17 | 0.39 | 0.7988 |

Table S5. Results of the statistical analysis of the effects of light conditions (two levels, only visible light /VIS/ or visible light supplemented with UV-A /VIS + UVA/) on the following properties measured for non-inoculated Arabidopsis plants on the 9-th day of growth: root system length (A), volume (B), average diameter (C). A linear model was fitted using the *gls* command of the *nlme* package in the R software. To control for heteroskedasticity, the model allowed for different variances in each group (*nlme* variance function *varIdent(form = ~ 1| light)*). Differences between cell means were examined using the *emmeans* package, with the approximate number of degrees of freedom calculated using Satterthwaite’s method.

**A**. Test of the difference in the mean root system length

| Hypothesis | Estimate  [mm] | SE [mm] | df | *t* | *p* |  |
| --- | --- | --- | --- | --- | --- | --- |
| (VIS + UVA) - (VIS) = 0 | -37.3 | 6.6 | 15.45 | -5.66 | 0.000041 | *** |

**B**. Test of the difference in the mean root system volume

| Hypothesis | Estimate [mm^3^] | SE [mm^3^] | df | *t* | *p* |  |
| --- | --- | --- | --- | --- | --- | --- |
| (VIS + UVA) - (VIS) = 0 | -0.41 | 0.6 | 15.62 | -0.68 | 0.5037 |  |

**C**. Test of the difference in the mean root diameter

| Hypothesis | Estimate [mm] | SE [mm] | df | *t* | *p* |  |
| --- | --- | --- | --- | --- | --- | --- |
| (VIS + UVA) - (VIS) = 0 | 0.036 | 0.02 | 13.91 | 1.81 | 0.0927 |  |

Table S6. The results of the statistical analysis of the effects of the light conditions (two levels: only visible light /VIS/, visible light supplemented with UV-A /VIS + UVA/), plant line (three levels: wild type, *fah1-2* and *tt4-11*) and inoculation status (two levels: inoculated vs non-inoculated) on the log-transformed ratio of fungal DNA to *Arabidopsis* DNA, measured with qPCR. Plants were inoculated with *Paraphoma chrysanthemicola* (A) or *Sporobolomyces ruberrimus* (B). A linear model of the relationship between the log-transformed endophyte DNA/plant DNA ratio and the predictors (light, plant line, inoculation status, their two- and three-way interactions, as well as the plant batch) was fitted using the *gls* command of the *nlme* package in the R software. To control for heteroskedasticity, the model allowed for variance to differ depending on inoculation status and light conditions (*nlme* variance function *varIdent(form = ~ 1|* *light * inoculationStatus)*). Differences between cell means of log-transformed responses were examined using the *emmeans* package, with the approximate number of degrees of freedom calculated using Satterthwaite’s method. *p* values were adjusted with Hommel’s method to keep the familywise error rate at 0.05. Four contrasts analyzed for the same endophyte were treated as a family for the purpose of the *p* adjustment.

**A**. Analysis of variance in the mean log-transformed ratio of *Paraphoma chrysanthemicola* DNA and *Arabidopsis* DNA levels

| Denominator df: 22 |  |  |  |  |  |  |  |
| --- | --- | --- | --- | --- | --- | --- | --- |
|  | Num. df | *F* | *p* |  |  |  |  |
| light | 1 | 0.98 | 0.3328 |  |  |  |  |
| inoculationStatus | 1 | 84.68 | 5.36·10^-9^ | *** |  |  |  |
| line | 2 | 1.19 | 0.3221 |  |  |  |  |
| batch | 2 | 1.54 | 0.2374 |  |  |  |  |
| light:inoculationStatus | 1 | 1.60 | 0.2197 |  |  |  |  |
| light:line | 2 | 1.15 | 0.3337 |  |  |  |  |
| line:inoculationStatus | 2 | 0.12 | 0.8891 |  |  |  |  |
| light:inoculationStatus:line | 2 | 0.15 | 0.8638 |  |  |  |  |

Tests of differences in the mean log-transformed ratio of *Paraphoma chrysanthemicola* DNA and

*Arabidopsis* DNA levels

| Hypothesis | Estimate | SE | df | *t* | *p_adjusted_* |  |
| --- | --- | --- | --- | --- | --- | --- |
| VIS : wt : mock-inoculated | -3.62 | 1.05 | 7.65 | -3.44 | 0.0095 | ** |
| VIS + UVA: wt : mock-inoculated | -5.57 | 1.54 | 8.38 | -3.62 | 0.0095 | ** |
| VIS:*fah1-2*:mock-inoculated | -4.43 | 1.05 | 7.65 | -4.21 | 0.0095 | ** |
| VIS + UVA:*fah1-2*:mock-inoculated | -6.00 | 1.54 | 8.38 | -3.90 | 0.0095 | ** |
| VIS:*tt4-21*:mock-inoculated | -4.75 | 1.05 | 7.65 | -4.51 | 0.0095 | ** |
| VIS + UVA:*tt4-21*:mock-inoculated | -5.32 | 1.54 | 8.38 | -3.46 | 0.0095 | ** |

**B**. Analysis of variance in the mean log-transformed ratio of *Sporobolomyces* *ruberrimus* DNA and *Arabidopsis* DNA levels

| Denominator df: 22 |  |  |  |  |  |  |  |
| --- | --- | --- | --- | --- | --- | --- | --- |
|  | Num. df | *F* | *p* |  |  |  |  |
| light | 1 | 0.0033 | 0.9545 |  |  |  |  |
| inoculationStatus | 1 | 79.43 | 9.40·10^-9^ | *** |  |  |  |
| line | 2 | 0.93 | 0.4083 |  |  |  |  |
| batch | 2 | 15.63 | 5.98·10^-5^ | *** |  |  |  |
| light: inoculationStatus | 1 | 0.006 | 0.9395 |  |  |  |  |
| light:line | 2 | 1.28 | 0.2986 |  |  |  |  |
| line:inoculationStatus | 2 | 0.65 | 0.5304 |  |  |  |  |
| light: inoculationStatus:line | 2 | 2.40 | 0.1144 |  |  |  |  |

Tests of differences in the mean log-transformed ratio of *Sporobolomyces* *ruberrimus* DNA and

*Arabidopsis* DNA levels

| Hypothesis | Estimate | SE | df | *t* | *p_adjusted_* |  |
| --- | --- | --- | --- | --- | --- | --- |
| VIS : wt : mock- inoculated | -2.16 | 0.40 | 6.52 | -5.36 | 0.0070 | ** |
| VIS + UVA: wt : mock -inoculated | -1.23 | 0.40 | 7.03 | -3.08 | 0.0351 | * |
| VIS:*fah1-2*: mock -inoculated | -1.22 | 0.40 | 6.52 | -3.04 | 0.0402 | * |
| VIS + UVA:*fah1-2*: mock -inoculated | -1.25 | 0.40 | 7.03 | -3.15 | 0.0324 | * |
| VIS:*tt4-21*: mock -inoculated | -1.03 | 0.40 | 6.52 | -2.55 | 0.0402 | * |
| VIS + UVA:*tt4-21*: mock -inoculated | -1.85 | 0.40 | 7.03 | -4.64 | 0.0117 | * |

Table S7. Results of the statistical analysis of the effects of endophyte inoculation (three levels, the mock-inoculated control and inoculation with either *Paraphoma* *chrysanthemicola* or *Sporobolomyces* *ruberrimus*), light conditions (two levels, only visible light /VIS/ or visible light supplemented with UV-A /VIS + UVA/), plant line (three levels, wild type, *tt4-11* and *fah1-2*) and their two- and three-way interactions on fresh shoot (A) and root (B) weight 128 h after inoculation, as well as the increase in the main root length within 100 h after inoculation (C). A linear model was fitted using the *lme* command of the *nlme* package in the R software. To control for heteroskedasticity, *nlme* variance function of the form *varIdent(form = ~ 1| endophyte*light)* was used. The batch of plants was treated as a random intercept factor. Differences between cell means were examined using the *emmeans* package, with the approximate number of degrees of freedom calculated using Satterthwaite’s method. *p* values were adjusted for the false discovery rate of 0.05 using the Benjamin-Hochberg method.

**A.** Analysis of variance of the fresh shoot weight measured 128 h after inoculation

| Denominator df:121 | | | |  |
| --- | --- | --- | --- | --- |
|  | Num. df | *F* | *p* |  |
| light | 1 | 10.76 | 0.0014 | ** |
| endophyte | 2 | 46.15 | 1.33·10^-15^ | *** |
| line | 2 | 8.59 | 0.0003 | *** |
| light:endophyte | 2 | 0.41 | 0.6652 |  |
| light:line | 2 | 0.27 | 0.7649 |  |
| endophyte:line | 4 | 0.60 | 0.6668 |  |
| light:endophyte:line | 4 | 0.42 | 0.7927 |  |

Tests of differences in the fresh shoot weight measured 128 h after inoculation

| Hypothesis | Estimate  [mg] | SE [mg] | df | *t* | *p_adjusted_* |  |
| --- | --- | --- | --- | --- | --- | --- |
| mock wt: VIS - (VIS + UVA) = 0 | 1.457 | 1.186 | 26.05 | 1.23 | 0.2932 |  |
| mock *fah1-2*: VIS - (VIS + UVA) = 0 | 1.712 | 1.186 | 26.05 | 1.44 | 0.2413 |  |
| mock *tt4-11*: VIS - (VIS + UVA) = 0 | 2.321 | 1.186 | 26.05 | 1.96 | 0.1168 |  |
| *Paraphoma* wt: VIS - (VIS + UVA) = 0 | 2.695 | 2.303 | 29.17 | 1.17 | 0.2932 |  |
| *Paraphoma* *fah1-2*: VIS - (VIS + UVA) = 0 | 2.347 | 2.303 | 29.17 | 1.02 | 0.3498 |  |
| *Paraphoma* *tt4-11*: VIS - (VIS + UVA) = 0 | 2.750 | 2.303 | 29.17 | 1.19 | 0.2932 |  |
| *Sporobolomyces* wt: VIS - (VIS + UVA) = 0 | 2.789 | 1.421 | 30.61 | 1.96 | 0.1168 |  |
| *Sporobolomyces* *fah1-2*: VIS - (VIS + UVA) = 0 | -0.139 | 1.563 | 28.49 | -0.09 | 0.9296 |  |
| *Sporobolomyces* *tt4-11*: VIS - (VIS + UVA) = 0 | 0.988 | 1.421 | 30.61 | 0.70 | 0.5166 |  |
| VIS wt: *Paraphoma* - mock = 0 | -2.915 | 1.930 | 24.39 | -1.51 | 0.2322 |  |
| VIS wt: *Sporobolomyces* - mock = 0 | -4.205 | 1.324 | 28.30 | -3.18 | 0.0151 | * |
| UV + VIS wt: *Sporobolomyces* - mock = 0 | -5.538 | 1.293 | 35.18 | -4.28 | 0.0014 | ** |
| UV + VIS wt: *Paraphoma* - mock = 0 | -4.153 | 1.728 | 33.58 | -2.40 | 0.0575 |  |
| VIS *fah1-2*: *Paraphoma* - mock = 0 | -4.500 | 1.930 | 24.39 | -2.33 | 0.0660 |  |
| VIS *fah1-2*: *Sporobolomyces* - mock = 0 | -6.994 | 1.476 | 26.00 | -4.74 | 0.0014 | ** |
| UV + VIS *fah1-2*: *Paraphoma* - mock = 0 | -5.134 | 1.728 | 33.58 | -2.97 | 0.0190 | * |
| UV + VIS *fah1-2*: *Sporobolomyces* - mock = 0 | -5.143 | 1.293 | 35.18 | -3.98 | 0.0023 | ** |
| VIS *tt4-11*: *Paraphoma* - mock = 0 | -2.614 | 1.930 | 24.39 | -1.35 | 0.2632 |  |
| VIS *tt4-11*: *Sporobolomyces* - mock = 0 | -4.940 | 1.324 | 28.30 | -3.73 | 0.0045 | ** |
| UV + VIS *tt4-11*: *Paraphoma* - mock = 0 | -3.042 | 1.728 | 33.58 | -1.76 | 0.1529 |  |
| UV + VIS *tt4-11*: *Sorobolomyces* - mock = 0 | -3.606 | 1.293 | 35.18 | -2.79 | 0.0254 | * |

**B.** Analysis of variance of the fresh root weight measured 128 h after inoculation

Denominator df:121

|  | Num. df | *F* | *p* |  |
| --- | --- | --- | --- | --- |
| light | 1 | 2.01 | 0.1588 |  |
| endophyte | 2 | 72.62 | >0.0001 | *** |
| line | 2 | 18.69 | >0.0001 | *** |
| light:endophyte | 2 | 0.93 | 0.3990 |  |
| light:line | 2 | 0.39 | 0.6751 |  |
| endophyte:line | 4 | 1.80 | 0.1324 |  |
| light:endophyte:line | 4 | 0.33 | 0.8238 |  |

Tests of differences in the fresh root weight measured 128 h after inoculation

| Hypothesis | Estimate  [mg] | SE [mg] | df | *t* | *p_adjusted_* |  | |
| --- | --- | --- | --- | --- | --- | --- | --- |
| mock wt: VIS - (VIS + UVA) = 0 | -0.345 | 1.276 | 35.59 | -0.27 | 0.8278 | |  |
| mock *fah1-2*: VIS - (VIS + UVA) = 0 | 0.7729 | 1.276 | 35.59 | 0.61 | 0.6774 | |  |
| mock *tt4-11*: VIS - (VIS + UVA) = 0 | 0.9692 | 1.276 | 35.59 | 0.76 | 0.6333 | |  |
| *Paraphoma* wt: VIS - (VIS + UVA) = 0 | 1.730 | 0.725 | 39.76 | 2.39 | 0.0460 | | * |
| *Paraphoma* *fah1-2*: VIS - (VIS + UVA) = 0 | 0.4471 | 0.725 | 39.76 | 0.62 | 0.6774 | |  |
| *Paraphoma* *tt4-11*: VIS - (VIS + UVA) = 0 | 1.3720 | 0.725 | 39.76 | 1.89 | 0.1065 | |  |
| *Sporobolomyces* wt: VIS - (VIS + UVA) = 0 | 0.241 | 1.413 | 39.13 | 0.17 | 0.8657 | |  |
| *Sporobolomyces* *fah1-2*: VIS - (VIS + UVA) = 0 | -0.801 | 1.508 | 39.83 | -0.53 | 0.6979 | |  |
| *Sporobolomyces* *tt4-11*: VIS - (VIS + UVA) = 0 | 0.674 | 1.413 | 39.13 | 0.48 | 0.7032 | |  |
| VIS wt: *Paraphoma* - mock = 0 | -4.730 | 0.887 | 34.18 | -5.33 | 4.41·10^-5^ | | *** |
| VIS wt: *Sporobolomyces* - mock = 0 | -3.401 | 1.181 | 37.54 | -2.88 | 0.0172 | | * |
| UV + VIS wt: *Paraphoma* - mock = 0 | -6.806 | 1.169 | 30.08 | -5.82 | 2.38·10^-5^ | | *** |
| UV + VIS wt: *Sporobolomyces* - mock = 0 | -3.986 | 1.493 | 39.18 | -2.67 | 0.0256 | | * |
| VIS *fah1-2*: *Paraphoma* - mock = 0 | -6.131 | 0.887 | 34.18 | -6.91 | 1.19·10^-6^ | | *** |
| VIS *fah1-2*: *Sporobolomyces* - mock = 0 | -4.514 | 1.294 | 34.92 | -3.49 | 0.0047 | | ** |
| UV + VIS *fah1-2*: *Paraphoma* - mock = 0 | -5.805 | 1.169 | 30.08 | -4.97 | 0.0001 | | *** |
| UV + VIS *fah1-2*: *Sporobolomyces* - mock = 0 | -2.940 | 1.493 | 39.18 | -1.97 | 0.0981 | |  |
| VIS *tt4-11*: *Paraphoma* - mock = 0 | -3.284 | 0.887 | 34.18 | -3.70 | 0.0032 | | ** |
| VIS *tt4-11*: *Sporobolomyces* - mock = 0 | -2.415 | 1.181 | 37.54 | -2.04 | 0.0917 | |  |
| UV + VIS *tt4-11*: *Paraphoma* - mock = 0 | -3.687 | 1.169 | 30.08 | -3.15 | 0.0109 | | * |
| UV + VIS *tt4-11*: *Sorobolomyces* - mock = 0 | -2.119 | 1.493 | 39.18 | -1.42 | 0.2455 | |  |

**C**. Analysis of variance of the increase of main root length within 100 h after inoculation

| Denominator df:123 | | | |  |
| --- | --- | --- | --- | --- |
|  | Num. df | *F* | *p* |  |
| light | 1 | 30.79 | 1.68·10^-7^ | *** |
| endophyte | 2 | 176.32 | 0 | *** |
| line | 2 | 3.46 | 0.0346 | * |
| light:endophyte | 2 | 5.95 | 0.0034 | ** |
| light:line | 2 | 1.01 | 0.3687 |  |
| endophyte:line | 4 | 0.23 | 0.9184 |  |
| light:endophyte:line | 4 | 1.45 | 0.2210 |  |

Tests of differences in the increase of the main root length within 100 h after inoculation

|  |  |  |  |  |  |  |  |
| --- | --- | --- | --- | --- | --- | --- | --- |
| Hypothesis | Estimate [cm] |  | SE  [cm] | df | *t* | *p_adjusted_* |  |
| mock wt: VIS - (VIS + UVA) = 0 | 1.247 |  | 0.338 | 39.12 | 3.69 | 0.0016 | ** |
| mock *fah1*-2: VIS - (VIS + UVA) = 0 | 1.398 |  | 0.338 | 39.12 | 4.14 | 0.0005 | *** |
| mock *tt4-11*: VIS - (VIS + UVA) = 0 | 1.000 |  | 0.338 | 39.12 | 2.96 | 0.0090 | ** |
| *Paraphoma* wt: VIS - (VIS + UVA) = 0 | 0.971 |  | 0.462 | 39.01 | 2.10 | 0.0553 |  |
| *Paraphoma* *fah1-2*: VIS - (VIS + UVA) = 0 | -0.270 |  | 0.462 | 39.01 | -0.58 | 0.5626 |  |
| *Paraphoma* *tt4-11*: VIS - (VIS + UVA) = 0 | 1.173 |  | 0.462 | 39.01 | 2.54 | 0.0229 | * |
| *Sporobolomyces* wt: VIS - (VIS + UVA) = 0 | 0.302 |  | 0.331 | 26.31 | 0.91 | 0.4328 |  |
| *Sporobolomyces* *fah1-2*: VIS - (VIS + UVA) = 0 | 0.224 |  | 0.331 | 26.31 | 0.68 | 0.5284 |  |
| *Sporobolomyces tt4-11*: VIS - (VIS + UVA) = 0 | 0.310 |  | 0.331 | 26.31 | 0.94 | 0.4328 |  |
| VIS wt: *Paraphoma* - mock = 0 | -1.357 |  | 0.426 | 35.27 | -3.19 | 0.0063 | ** |
| VIS wt: *Sporobolomyces* - mock = 0 | -2.933 |  | 0.377 | 36.82 | -7.77 | 2.96·10^-8^ | *** |
| UV + VIS wt: *Paraphoma* - mock = 0 | -1.081 |  | 0.382 | 35.34 | -2.83 | 0.0124 | * |
| UV + VIS wt: *Sporobolomyces* - mock = 0 | -1.987 |  | 0.285 | 34.87 | -6.97 | 1.48·10^-7^ | *** |
| VIS *fah1-2*: *Paraphoma* - mock = 0 | -1.970 |  | 0.426 | 35.27 | -4.62 | 0.0001 | *** |
| VIS *fah1-2*: *Sporobolomyces* - mock = 0 | -3.293 |  | 0.377 | 36.82 | -8.72 | 3.60·10^-9^ | *** |
| UV + VIS *fah1-2*: *Paraphoma* - mock = 0 | -0.302 |  | 0.382 | 35.34 | -0.79 | 0.4812 |  |
| UV + VIS *fah1-2*: *Sporobolomyces* - mock = 0 | -2.120 |  | 0.285 | 34.87 | -7.44 | 4.52·10^-8^ | *** |
| VIS *tt4-11*: *Paraphoma* - mock = 0 | -1.016 |  | 0.426 | 35.27 | -2.38 | 0.0316 | * |
| VIS *tt4-11*: *Sporobolomyces* - mock = 0 | -2.859 |  | 0.377 | 36.82 | -7.58 | 3.42·10^-8^ | *** |
| UV + VIS *tt4-11*: *Paraphoma* - mock = 0 | -1.189 |  | 0.382 | 35.34 | -3.11 | 0.0070 | ** |
| UV + VIS *tt4-11*: *Sorobolomyces* - mock = 0 | -2.169 |  | 0.285 | 34.87 | -7.61 | 3.42·10^-8^ | *** |
