# Supplementary figures and images for: Effect of UV-A on endophyte colonisation of Arabidopsis thaliana
Source: PLoS One. 2025 May 15;20(5):e0323576. doi: 10.1371/journal.pone.0323576 (PMC12080771; doi:10.1371/journal.pone.0323576)

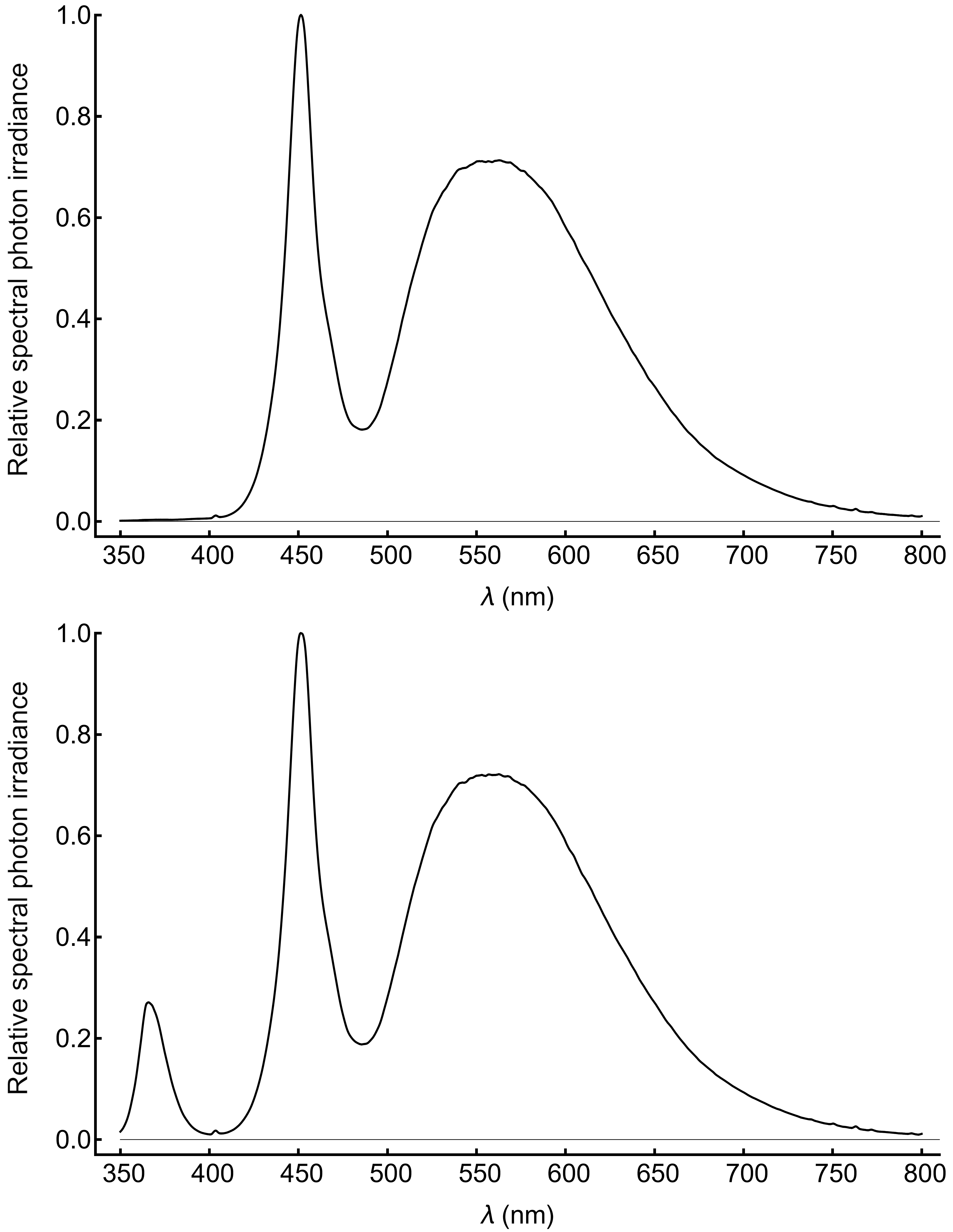

Supplement: S1 Fig — White illumination of ca. 225 μmol m-2 s-1 PAR was provided by LEDs (EPILEDs, 5000 K) supplemented with UV-A of 13 μmol m-2 s-1 from fluorescent tubes (Philips TL 6W BLB) covered with either Lee #226 gel filter (upper panel), or with a polyester film, Autostat CT5 (lower panel). (TIFF) [file pone.0323576.s003.tiff]

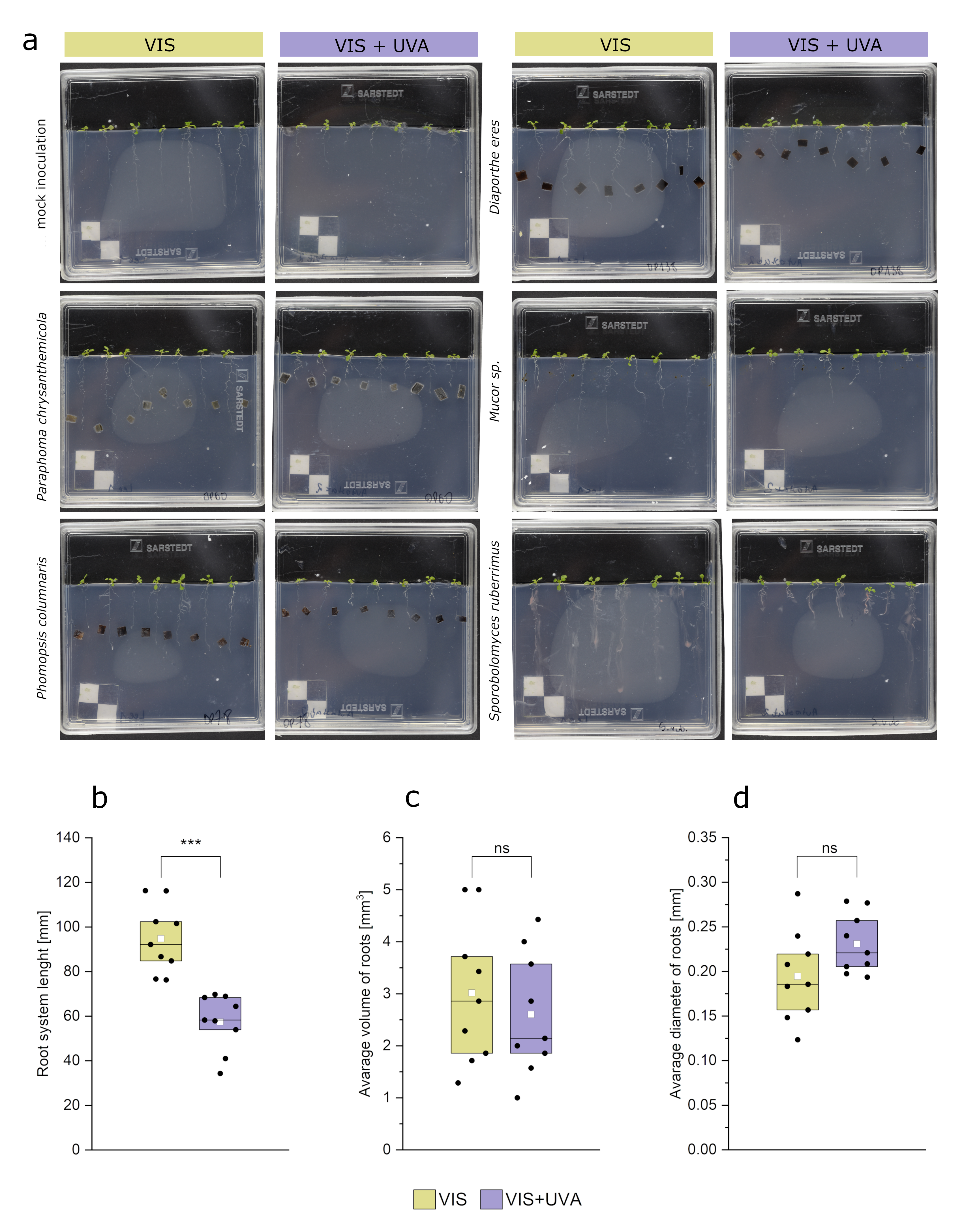

Supplement: S2 Fig — (b - d) Length (b), average volume (c), and average diameter (d) of roots of non-inoculated plants on the 9th day of growth. The experiment consists of nine biological replicates (plates) per every combination of light conditions and inoculation type. In b, the bracket shows a significant difference in means (*** p ≤ 0.001). The results of the statistical analysis are in the S2 Appendix, Table S5. White and black rectangles are 1 cm by 1 cm each. (TIF) [file pone.0323576.s004.tif]

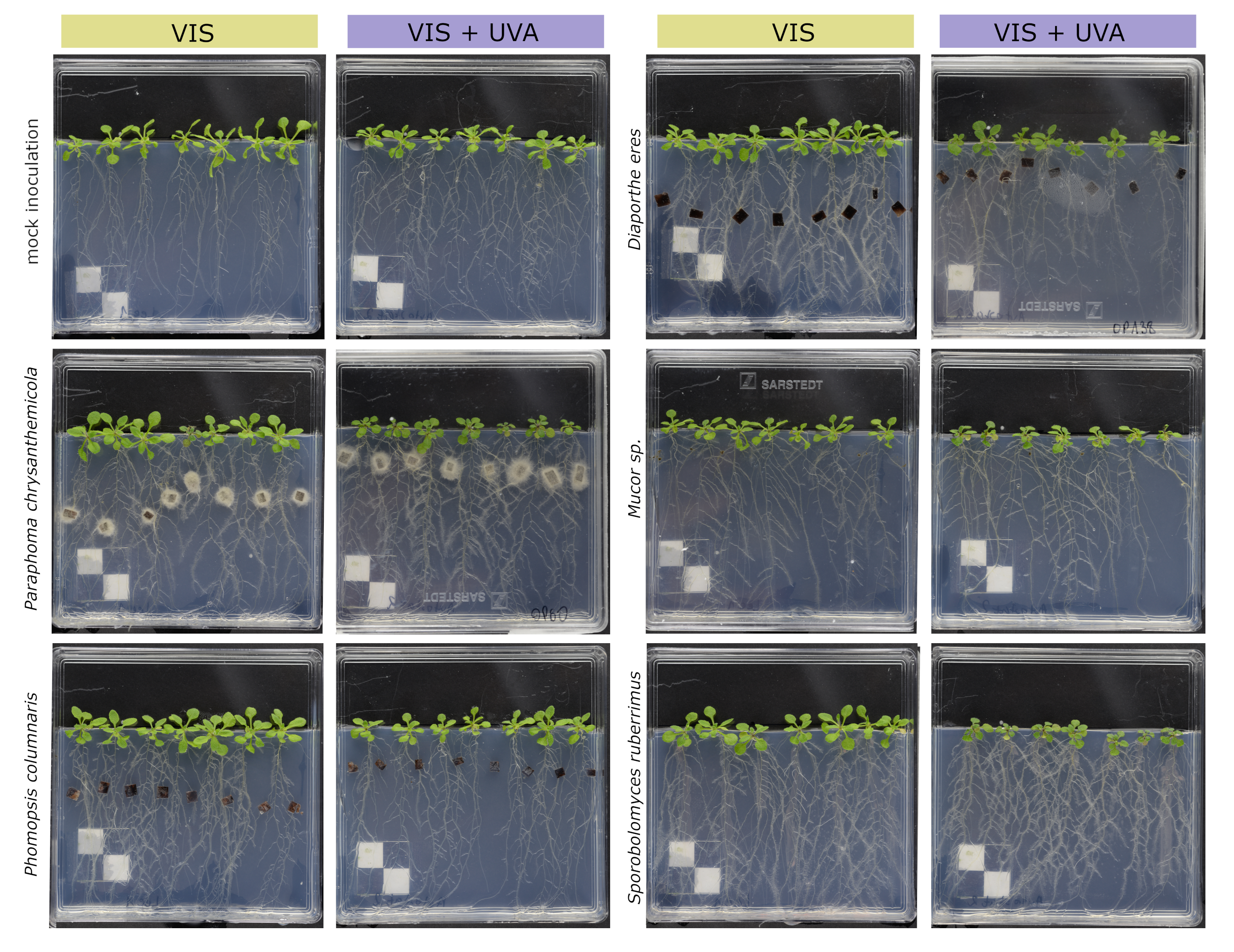

Supplement: S3 Fig — White and black rectangles are 1 cm by 1 cm each. (TIF) [file pone.0323576.s005.tif]

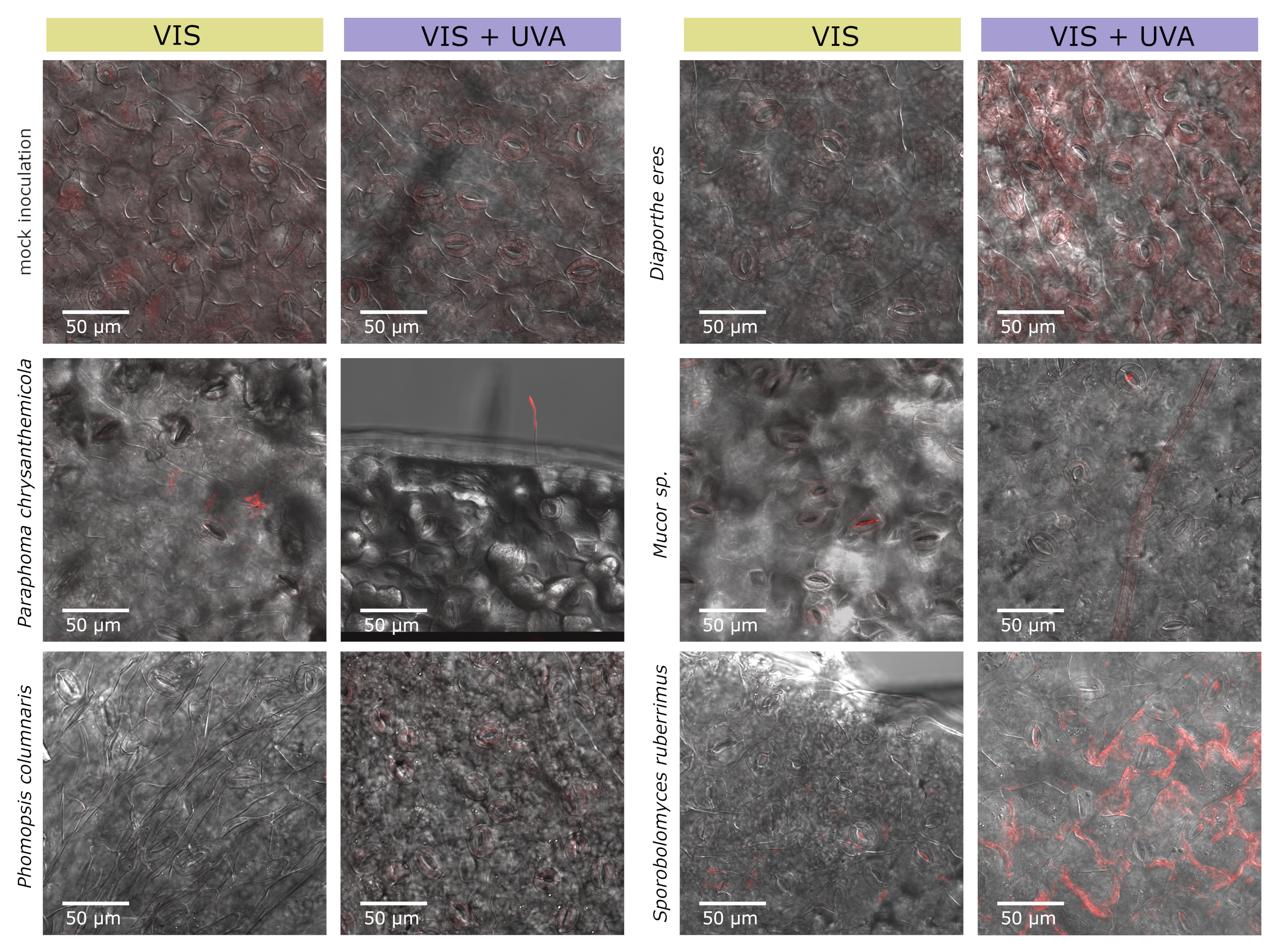

Supplement: S4 Fig — Endophytes were stained Wheat Germ Agglutinin conjugated with Texas Red. Images, recorded with a laser scanning confocal microscope, show transmitted light channel merged with a red fluorescence channel. Plants not inoculated with the endophyte serve as a control for plant tissue autofluorescence. Scale bars = 50 μm. (TIF) [file pone.0323576.s006.tif]

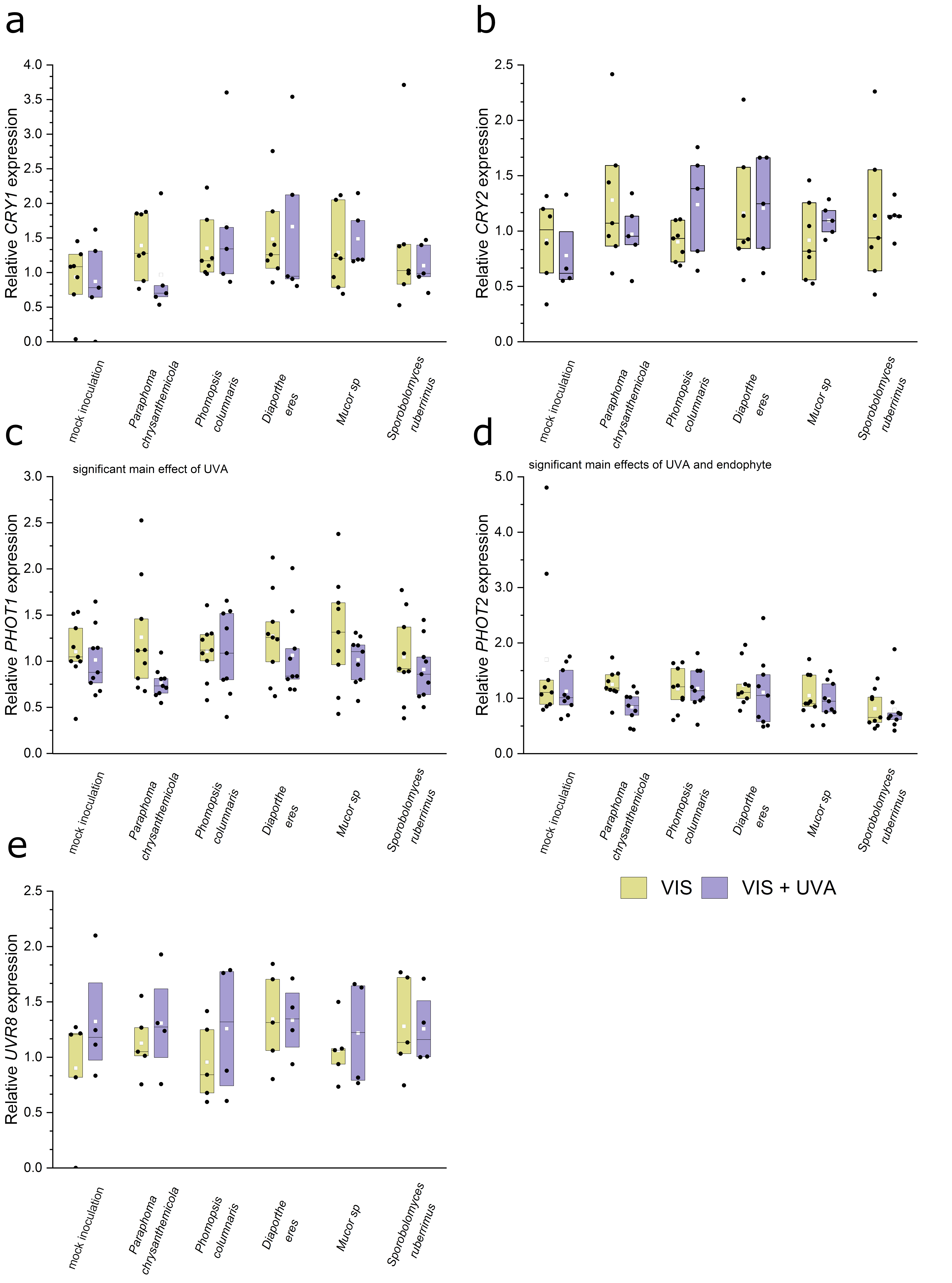

Supplement: S5 Fig — The experiment was performed in nine biological replicates for PHOT1, and PHOT2 and six biological replicates for CRY1, CRY2, and UVR8. The results of the statistical analysis can be found in the S2 Appendix, Table S3. (TIF) [file pone.0323576.s007.tif]

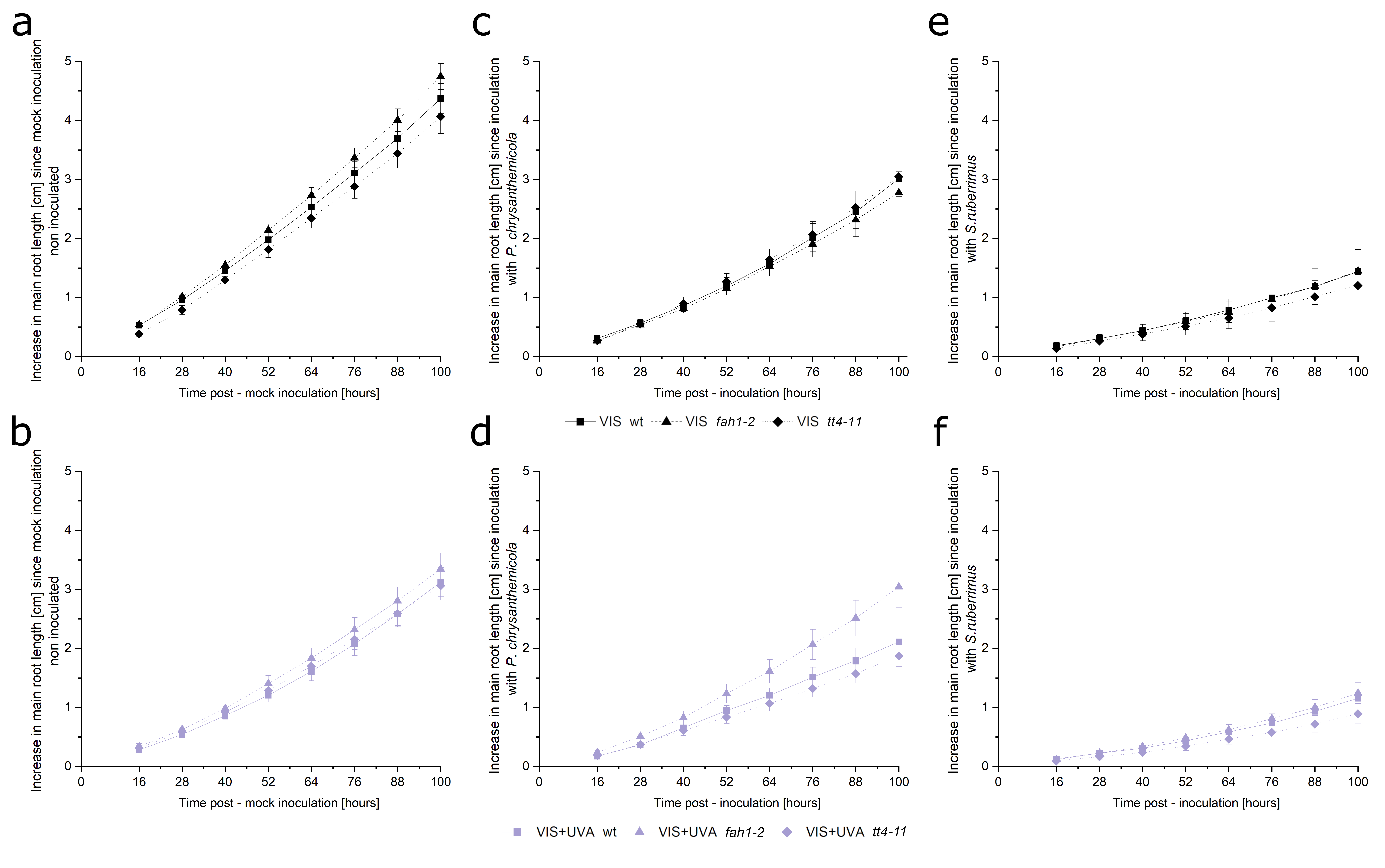

Supplement: S6 Fig — Plants were grown in the presence or absence of UV-A. The experiment was performed in 8 biological replicates (independent plates) for every combination of inoculation type and light conditions. (TIF) [file pone.0323576.s008.tif]
