# Supplementary material for: Effect of UV-A on endophyte colonisation of Arabidopsis thaliana
Source: PLoS One. 2025 May 15;20(5):e0323576. doi: 10.1371/journal.pone.0323576 (PMC12080771; doi:10.1371/journal.pone.0323576)
Supplement: Attachment — Submitted filename: review-PLoS One.docx [file pone.0323576.s009.docx]

This study investigates the effects of UV-A radiation on the interaction between Arabidopsis thaliana and endophytic fungi. The overall experimental design is well-structured and the data is rich; however, there are several areas that could be improved. Below are specific questions and suggestions for revision, concerning the experimental design, data analysis, discussion, and other aspects, aiming to enhance the quality of the research and the paper:

1. Although the proportion of UV-A radiation to Photosynthetically Active Radiation (PAR) was set to closely resemble natural sunlight, other environmental conditions in the growth chamber may still differ significantly from natural environments. For example, factors such as temperature fluctuations, humidity variations, and the combined effect of other environmental factors were not adequately simulated in the experiment. These differences could affect the generalizability of the results. It is recommended that the authors discuss how these discrepancies may influence the plant-endophyte interaction or supplement the study with experiments conducted under more natural environmental conditions to enhance the applicability of the conclusions.

2. There are differences in the inoculation methods and concentrations used for different endophytes. For instance, Paraphoma chrysanthemicola, Phomopsis columnaris, and Diaporthe eres were inoculated using mycelial blocks, while Mucor sp. was inoculated with spores, and Sporobolomyces ruberrimus was inoculated with yeast cell suspensions. Additionally, the specific inoculation quantities are described inconsistently. These variations may interfere with the accurate comparison of the effects of UV-A on different endophytes. It is suggested that the authors standardize the inoculation concentration reporting methods, or conduct additional experiments to investigate the influence of different inoculation concentrations on the results, in order to confirm the reliability of the current inoculation methods and concentrations.

3. In the discussion section, some interpretations of the results are inconsistent with previous studies. For example, in interpreting the effects of UV-A on plant growth, there are discrepancies between the current findings and those in other studies regarding the promotion or inhibition of plant growth by UV-A. However, the authors did not thoroughly explore the reasons for these differences.

4. Based on the experimental results, the authors may consider constructing a conceptual model to integrate the interactions between UV-A, endophytes, and plants. This would help to clarify the relationships between the different factors and how they collectively influence plant growth, defense mechanisms, and metabolic processes.

5. Given that the study involves plant-microbe interactions in agriculture and the impact of UV-A radiation, it is recommended that the authors further explore the practical applications of their findings in agricultural production. For example, how you results might be used to optimize greenhouse cultivation conditions or how an understanding of the relationship between UV-A and endophytes could contribute to the development of new agricultural strategies to improve crop yield and quality, thereby increasing the practical relevance of the study.

6. The UV-A radiation intensity used in the study was 13 µmol m⁻² s⁻¹, but the rationale for selecting this specific intensity was not explained. The authors are encouraged to provide a justification for this choice, indicating whether it corresponds to natural UV-A intensity levels or if there is supporting literature that highlights the physiological relevance of this particular intensity.

7. While the paper measures parameters such as fresh weight, root length, and root volume, there is limited discussion of how changes in these parameters may be related to endophyte colonization or UV-A radiation. It is recommended that the authors conduct a more in-depth analysis of whether these parameter changes are directly associated with endophytic colonization or UV-A exposure and provide more physiological explanations for these observations.
